# Characterization of antimicrobial-resistant Gram-negative bacteria that cause neonatal sepsis in seven low- and middle-income countries

**DOI:** 10.1038/s41564-021-00870-7

**Published:** 2021-03-29

**Authors:** Kirsty Sands, Maria J. Carvalho, Edward Portal, Kathryn Thomson, Calie Dyer, Chinenye Akpulu, Robert Andrews, Ana Ferreira, David Gillespie, Thomas Hender, Kerenza Hood, Jordan Mathias, Rebecca Milton, Maria Nieto, Khadijeh Taiyari, Grace J. Chan, Delayehu Bekele, Semaria Solomon, Sulagna Basu, Pinaki Chattopadhyay, Suchandra Mukherjee, Kenneth Iregbu, Fatima Modibbo, Stella Uwaezuoke, Rabaab Zahra, Haider Shirazi, Adil Muhammad, Jean-Baptiste Mazarati, Aniceth Rucogoza, Lucie Gaju, Shaheen Mehtar, Andre N. H. Bulabula, Andrew Whitelaw, BARNARDS Group, Timothy R. Walsh

**Affiliations:** 1 Division of Infection and Immunity, Cardiff University, Cardiff, UK; 2 Department of Zoology, University of Oxford, Oxford, UK; 3 Institute of Biomedicine, Department of Medical Sciences, University of Aveiro, Aveiro, Portugal; 4 Centre for Trials Research, Cardiff University, Cardiff, UK; 5 National Hospital Abuja, Abuja, Nigeria; 6 54gene, Lagos, Nigeria; 7 Division of Medical Critical Care, Boston Children’s Hospital, Boston, MA USA; 8 Department of Pediatrics, Harvard Medical School, Boston, MA USA; 9 Department of Epidemiology, Harvard T.H. Chan School of Public Health, Boston, MA USA; 10 Department of Obstetrics and Gynecology, St Paul’s Hospital Millennium Medical College, Addis Ababa, Ethiopia; 11 Department of Microbiology, Immunology and Parasitology, St Paul’s Hospital Millennium Medical College, Addis Ababa, Ethiopia; 12 Division of Bacteriology, ICMR–National Institute of Cholera and Enteric Diseases, Kolkata, India; 13 Department of Neonatology, Institute of Postgraduate Medical Education & Research, Kolkata, India; 14 Federal Medical Centre – Jabi, Abuja, Nigeria; 15 Department of Microbiology, Quaid-i-Azam University, Islamabad, Pakistan; 16 Pakistan Institute of Medical Sciences, Islamabad, Pakistan; 17 The National Reference Laboratory, Rwanda Biomedical Centre, Kigali, Rwanda; 18 Unit of IPC, Stellenbosch University, Cape Town, South Africa; 19 Infection Control Africa Network, Cape Town, South Africa; 20 Department of Global Health, Stellenbosch University, Cape Town, South Africa; 21 Division of Medical Microbiology, Stellenbosch University, Cape Town, South Africa; 22 National Health Laboratory Service, Tygerberg Hospital, Cape Town, South Africa; 23 https://www.barnards-group.com; 24 Ineos Oxford Institute for Antimicrobial Research, Department of Zoology, Oxford, UK

**Keywords:** Genomics, Antimicrobial resistance, Bacteriology

## Abstract

Antimicrobial resistance in neonatal sepsis is rising, yet mechanisms of resistance that often spread between species via mobile genetic elements, ultimately limiting treatments in low- and middle-income countries (LMICs), are poorly characterized. The Burden of Antibiotic Resistance in Neonates from Developing Societies (BARNARDS) network was initiated to characterize the cause and burden of antimicrobial resistance in neonatal sepsis for seven LMICs in Africa and South Asia. A total of 36,285 neonates were enrolled in the BARNARDS study between November 2015 and December 2017, of whom 2,483 were diagnosed with culture-confirmed sepsis. *Klebsiella pneumoniae* (*n* = 258) was the main cause of neonatal sepsis, with *Serratia marcescens* (*n* = 151), *Klebsiella michiganensis* (*n* = 117), *Escherichia coli* (*n* = 75) and *Enterobacter cloacae* complex (*n* = 57) also detected. We present whole-genome sequencing, antimicrobial susceptibility and clinical data for 916 out of 1,038 neonatal sepsis isolates (97 isolates were not recovered from initial isolation at local sites). Enterobacterales (*K. pneumoniae, E. coli* and *E. cloacae*) harboured multiple cephalosporin and carbapenem resistance genes. All isolated pathogens were resistant to multiple antibiotic classes, including those used to treat neonatal sepsis. Intraspecies diversity of *K. pneumoniae* and *E. coli* indicated that multiple antibiotic-resistant lineages cause neonatal sepsis. Our results will underpin research towards better treatments for neonatal sepsis in LMICs.

## Abstract (web summary)

Genomic and clinical analysis of 916 bacterial isolates from neonates with sepsis in seven low- and middle-income countries (the BARNARDS study) reveals that the main species present were antimicrobial-resistant *Klebsiella*, *Escherichia coli* and *Enterobacter*.

## Main

Although there has been a substantial decrease in infant mortality over the past 20 years^1^, the burden remains substantial, with the mortality rate of children under 5 years of age at 38 per 1,000 live births in 2019^2^, and 98% of recorded neonatal deaths now occurring in LMICs^3^. The World Health Organization (WHO) has declared neonatal sepsis a global concern^4^ and the burden of neonatal infectious diseases a major challenge. Effective management of sepsis is not always possible when resources are limited^3^, and the steady increase of antimicrobial resistance (AMR) worldwide further compromises sepsis management^5,6^.

Despite the burden of neonatal sepsis, accurate information on the causes and consequences of neonatal sepsis in LMICs is scarce^4,6^. Most studies in LMICs are from a single site, are of limited sample size, or lack accurate methods for sepsis diagnosis, pathogen identification and antibiotic susceptibility measurements^7–10^. In 2015 and 2016, two multicentre neonatal sepsis studies in LMICs were published^11,12^. However, neither study combined antimicrobial susceptibility testing and whole-genome sequencing (WGS), making it difficult to determine the extent of genomic diversity (which would usually be done by comparing the lineages across geographical areas) and resistance. The studies that have taken this approach were of single sites and often used WGS to investigate specific outbreaks^13–16^.

In LMICs, the epidemiology of early-onset sepsis (EOS) and late-onset sepsis (LOS) is not well defined^3^, unlike in high-income countries, where group B *Streptococcus* is usually considered the main cause of EOS^17^. A systematic review of the causes of blood culture-positive neonatal sepsis in Sub-Saharan Africa by Okomo et al.^18^ found that *Klebsiella* species, *Escherichia coli, Enterobacter* species and *Pseudomonas* species accounted for 38% of cases. Other single-site reports showed concordant findings^9,11,12,19^. However, these studies did not specifically determine whether certain species, or sequence type (ST) groups, are more likely to harbour resistance or virulence determinants, how this compares between different geographical areas and whether there is any relation to sepsis onset or outcome.

Burden of Antibiotic Resistance in Neonates from Developing Societies (BARNARDS; www.barnards-group.com) is a network of 12 clinical study sites in four African (Ethiopia, Nigeria, Rwanda and South Africa) and three South Asian countries (Bangladesh, India and Pakistan). The aim of the BARNARDS study is to assess the burden of AMR in neonates in these LMIC. Here, we report on the isolation and characterization of Gram-negative bacteria (GNB) causing neonatal sepsis in seven LMICs, including their AMR profiles. We report associations between phenotypic and genotypic data and sepsis onset and mortality following biological sepsis (MFBS). We also analyse whole-genome sequences from isolates that cause neonatal sepsis.

## Results

### Enrolment in BARNARDS and isolation of pathogenic bacteria

**Fig. 1:**
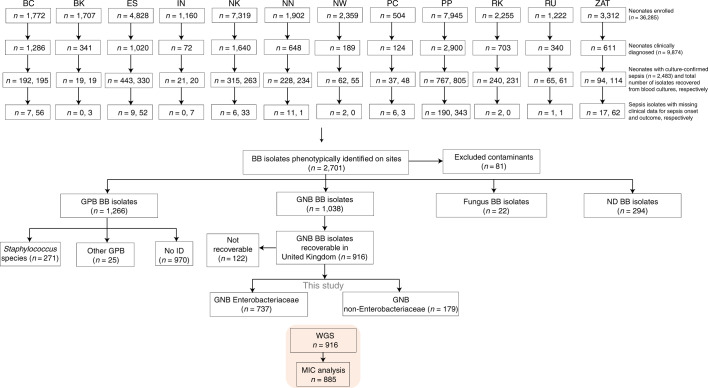
Flow chart detailing the enrolment of neonates and incidence of sepsis (both clinically diagnosed and confirmed by culture) per site. The numbers of neonates with missing clinical data for the onset and outcome of sepsis are shown per site. The numbers of isolates collected from neonatal blood cultures are shown per site, with a breakdown by preliminary characterization (as determined by Gram stain) in the flow chart below. The final numbers of isolates included for analysis are highlighted in orange. BB, baby blood; ID, identification (of species); ND, not determined.

**Fig. 2:**
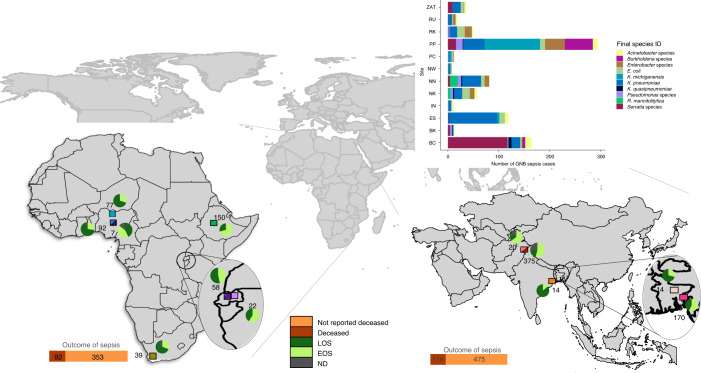
World map showing the BARNARDS study clinical site locations. The study sites are indicated by coloured squares. The African sites were located in Ethiopia (ES (green)), Nigeria (NK (cyan), NN (light blue) and NW (dark blue)), Rwanda (RK (dark purple) and RU (light purple)) and South Africa (ZAT; olive). The Asian sites were located in Bangladesh (BC (dark pink) and BK (light pink)), India (IN (orange)) and Pakistan (PC (peach) and PP (burgundy)). The numbers next to each clinical site location represent the total number of GNB identified. Inset: the stacked bar graph shows the distribution of the top ten GNB species recovered from blood cultures at the local sites. The onset of neonatal sepsis (EOS, LOS or ND) for GNB per clinical site is represented as a pie chart. The outcome of neonatal sepsis is shown for each continent.

The numbers of neonates recruited, clinically diagnosed with sepsis and with a confirmation of sepsis by positive blood culture are outlined in Fig. 1. Of 36,285 infants ≤60 d old (termed herein as neonates) enrolled in the BARNARDS study from November 2015 to December 2017, 2,483 had culture-confirmed sepsis. All 12 clinical sites used the same criteria for clinical diagnosis of sepsis (Supplementary Fig. 1). We found that cases were mainly EOS for both sites in Pakistan, one site in Bangladesh (BC; see Methods for definitions of all two-letter site abbreviations) and the single site in Ethiopia. In Nigeria, India and South Africa, there were mainly LOS cases. In Rwanda, neonatal sepsis cases were equally split between EOS and LOS (Fig. 2).

Automated blood culture systems were used to detect microbial growth, with 2,620 microbial isolates recovered. These 2,620 isolates comprised 1,266 Gram-positive bacteria (GPB) isolates, 1,038 GNB isolates, 22 fungal isolates and 294 unassigned isolates (Fig. 1). The methods for collection and identification were standardized across all sites, with equipment and reagents purchased from uniform suppliers. The primary aim of the BARNARDS study was to characterize the extent of β-lactam resistance in GNB causing clinically diagnosed sepsis in infants <60 d old (see Supplementary Figs. 2 and 3). However, at month 17 (during a BARNARDS network event), we anecdotally noted high rates of isolation of *Staphylococcus* species and therefore collected all of these isolates for analysis (to be reported elsewhere). Of 1,038 isolates, 916 GNB were analysed using WGS. For 122 isolates, identification beyond a Gram stain was not possible because the isolate was lost and/or purification for DNA extraction was unsuccessful (Fig. 1).

In total, 58 different species of GNB were identified across all sites by WGS (Supplementary Table 1), including *K. pneumoniae* (*n* = 258), *Serratia marcescens* (*n* = 151), *Klebsiella michiganensis* (*n* = 117), *Enterobacter* species (*n* = 80), *E. coli* (*n* = 75), *Burkholderia* species (*n* = 61)*, Acinetobacter* species (*n* = 49), *Pseudomonas* species (*n* = 36) and *Ralstonia mannitolilytica* (*n* = 21) (Fig. 2). Among the GNB characterized herein, 401 were *Klebsiella*, with six species identified: *K. pneumoniae*, *K. variicola, K. quasipneumoniae, K. aerogenes, K. oxytoca* and *K. michiganensis* (Supplementary Fig. 4). *Burkholderia* *cenocepacia, K. michiganensis, R. mannitolilytica* and *S. marcescens* were mainly isolated from samples obtained from single sites in Pakistan, Nigeria and Bangladesh (Fig. 2).

Overall, *R. mannitolilytica, K. michiganensis, Burkholderia* species and *Pseudomonas* species caused more cases of EOS than other species (Supplementary Table 2 and Supplementary Fig. 5). Of note, similar proportions of fatal sepsis cases were caused by GNB on each continent (92/353 cases in Africa (21%) and 118/475 cases in Asia (20%)). We found that *Burkholderia* species or *K. michiganensis* sepsis infections were the most likely to be fatal (Supplementary Table 2 and Supplementary Fig. 5). However, there was a large proportion of missing data from certain clinical sites (due to data collection and entry error; Fig. 1), which limited the strength of conclusions.

### Interspecies and intraspecies diversity across clinical sites

To understand the extent of inter- and intraspecies diversity, we aimed to perform WGS on all GNB. Multilocus sequence typing (MLST) was used primarily as an unambiguous tool to examine bacterial relatedness. As MLST can be performed easily via PCR of housekeeping genes, in addition to in silico MLST via WGS, assessing relatedness via this tool (where applicable) provides a dataset that can be extrapolated to existing data on neonatal sepsis where WGS may not be available.

MLST revealed substantial intraspecies diversity, and 40 previously unknown STs were assigned in 12 species (Table 1 and Supplementary Table 2). Fourteen STs were assigned in the *Klebsiella* genera (Table 1), including all three *K. aerogenes* STs (all from Africa; *n* = 2 from Nigeria and *n* = 1 from South Africa; ST194–196). *K. michiganensis* was mainly ST180 (from PP in Pakistan; Table 1). Such low ST diversity and close phylogenetic relatedness, as shown by the core genome phylogeny (Supplementary Fig. 6), warrant further investigation. Similarly, we noticed that a single, previously unknown, *B. cenocepacia* ST (ST1621), also from PP, was dominant (Table 1 and Supplementary Fig. 7). *B. cenocepacia* ST1621 from PP and the *S. marcescens* isolates from BC were indistinguishable during core genome analysis (Supplementary Figs. 7 and 8). All local-level clusters will be studied further.

**Table 1:** STs for the most commonly identified species with a recognized MLST scheme

Species	Isolates characterized by WGS^a^	Number identified per clinical site^b^	Number of STs found	Prominent STs	STs predominantly found in specific clinical sites	Previously unknown STs
*A. baumannii* complex	41 (38 ABA and 3 ANO)	BC (*n* = 8), BK (*n* = 2), ES (*n* = 7), IN (*n* = 3), NK (*n* = 3), PC (*n* = 1), PP (*n* = 8), RK (*n* = 2), RU (*n* = 3) and ZAT (*n* = 3)	15	ST1, ST2, ST575 and ST1106	ST2 (BC) and ST1106 (PP)	ST1326 and ST1327
*B. cenocepacia*	56	BC (*n* = 1), PC (*n* = 1) and PP (*n* = 54)	5	ST1621	ST1621 (PP)	ST1621 and ST1623
*E. coli*	75	BC (*n* = 3), ES (*n* = 11), IN (*n* = 2), NK (*n* = 15), NN (*n* = 7), NW (*n* = 1), PC (*n* = 3), PP (*n* = 10), RK (*n* = 15), RU (*n* = 2) and ZAT (*n* = 6)	37	ST10, ST69, ST131, ST410 and ST517	ST410 (PC) and ST517 (RK)	ND
*E. cloacae* complex	78 (1 EAS, 57 ECL, 18 EHO, 1 EKO and 1 ELU)	BC (*n* = 2), NK (*n* = 7), NN (*n* = 9), NW (*n* = 1), PP (*n* = 39), RK (*n* = 14), RU (*n* = 5) and ZAT (*n* = 1)	34	ST84, ST93, ST171, ST346, ST523 and ST980	ST84 (PP), ST93 (PP), ST171 (PP), ST346 (RK), ST523 (PP) and ST980 (RK)	ST1236 and ST1238–ST1248
*K. michiganensis*/ *K. oxytoca*	122 (117 KMI and 5 KOX)	ES (*n* = 5), PC (*n* = 4), PP (*n* = 111) and RK (*n* = 2)	5	ST180	ST180 (PP)	ST268 (KMI) and ST243–ST244 (KOX)
*K. pneumoniae*	258	BC (*n* = 17), BK (*n* = 2), ES (*n* = 95), IN (*n* = 5), NK (*n* = 16), NN (*n* = 37), NW (*n* = 4), PC (*n* = 2), PP (*n* = 42), RK (*n* = 15), RU (*n* = 7) and ZAT (*n* = 16)	57	ST15, ST35, ST37, ST39, ST218, ST307, ST348, ST443, ST464 and ST985	ST15 (PP), ST35 (ES), ST37 (ES), ST218 (ES), ST307 (RK), ST442 (NN), ST464 (NN) and ST985 (ES)	ST4008, ST4410 and ST4411
*K. quasipneumoniae*	13	BC (*n* = 6), BK (*n* = 1), ES (*n* = 1), NK (*n* = 2), NN (*n* = 2) and PP (*n* = 1)	10	ST4405	ST4405 (BC)	ST4405–ST4407 and ST4409
*K. variicola*	5	BC (*n* = 1), PP (*n* = 1), RK (*n* = 2) and RU (*n* = 1)	5	ND	ND	ST4404 and ST4412–ST4414
*P. aeruginosa*	23	BC (*n* = 1), BK (*n* = 4), IN (*n* = 1), NK (*n* = 2), NN (*n* = 3) PC (*n* = 1), PP (*n* = 9) and RK (*n* = 2)	14	ST3235, ST1285 and ST3311	ST1285 (BK) and ST3311 (PP)	ST3311
*Salmonella enterica*	7	NK (*n* = 6) and NN (*n* = 1)	4	ST313	ST313 (NK)	ND

^a^Numbers of isolates characterized. For complexes, a breakdown by species is given.

^b^Numbers of isolates from each clinical site.

ABA, *A. baumannii*; ANO, *A. nosocomialis*; EAS, *Enterobacter asburiae*; ECL, *E. cloacae*; EHO, *Enterobacter hormaechei*; EKO, *Enterobacter kobei*; ELU, *Enterobacter ludwigii*; KMI, *K. michiganensis*; KOX, *K. oxytoca*.

*Enterobacter cloacae* complex isolates were identified belonging to *E. cloacae*, *E. hormaechei*, *E. kobei*, *E. asburiae* and *E. ludwigii*. In total, seven different *Enterobacter* species (*n* = 80) with 28 STs were identified. The majority of *Enterobacter* species were found in Pakistan (*n* = 39), Nigeria (*n* = 19) and Rwanda (*n* = 14) (Fig. 1 and Supplementary Fig. 9). ST171 was common across sites in Africa and Asia; however, ST346 was only detected in Rwanda, ST523 was only detected in Pakistan and ST850 was only detected in Nigeria. Within *Enterobacter* species, 13 STs were assigned (Table 1) to *E. cloacae*, *E. hormaechei* and *E. ludwigii* isolates.

*Acinetobacter* species were recovered from ten out of 12 clinical sites in both Africa and South Asia, and 38 out of 49 (78%) were *Acinetobacter baumannii*. Of these, 17 out of 38 (45%) belonged to international clones (Pasteur MLST) ST1 and ST2 (Supplementary Tables 1 and 2).

### AMR of pathogens causing neonatal sepsis

One aim of the BARNARDS study was to describe the AMR profiles of pathogens causing neonatal sepsis. For this, we performed agar dilution to determine the minimum inhibitory concentrations (MICs) of 19 antibiotics, including the current recommended first-line empirical treatments for neonatal sepsis, as well as carbapenems (the incidence of carbapenem-resistant GNB is increasing at an alarming rate), on 885 GNB (31 isolates were not recovered following storage at −80 °C after genomic DNA extraction for WGS; Supplementary Table 2 lists the isolates recoverable for WGS and/or MIC testing). Current data in LMICs focus on profiling AMR within certain species only, and are therefore not exhaustive across all pathogens causing sepsis.

**Fig. 3:**
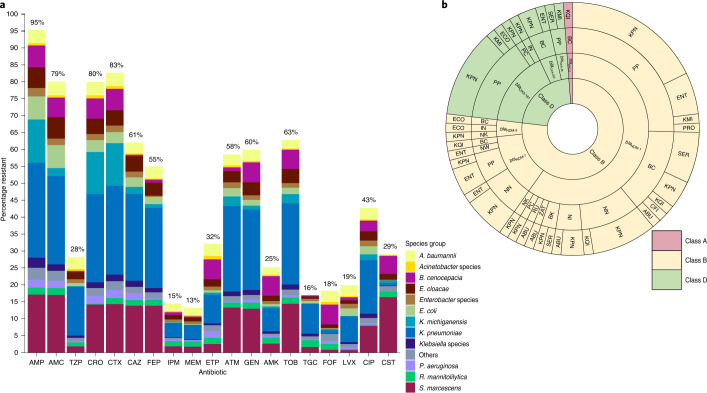
AMR of neonatal sepsis-causing pathogens. **a**, Percentages of antimicrobial-resistant aetiological agents of neonatal sepsis, coloured according to bacterial species/group (*n* = 885 isolates of GNB). The MICs of the antibiotics were determined by agar dilution and the results were interpreted according to EUCAST guidelines and documents^20,21^. AMC, amoxicillin/clavulanate; AMK, amikacin; AMP, ampicillin; ATM, aztreonam; CAZ, ceftazidime; CIP, ciprofloxacin; CRO, ceftriaxone; CST, colistin; CTX, cefotaxime; ETP, ertapenem; FEP, cefepime; FOF, fosfomycin; GEN, gentamicin; IPM, imipenem; LVX, levofloxacin; MEM, meropenem; TGC, tigecycline; TOB, tobramycin; TZP, piperacillin/tazobactam. **b**, Sunburst diagram detailing the class A (red), B (yellow) and D (green) carbapenemase resistance genes detected. The second ring from the centre shows the carbapenemase genes identified. The distributions across species and clinical sites are shown in the outer rings. ABU, *Acinetobacter baumannii*; CFI, *Citrobacter freundii*; ECO, *Escherichia coli*; ENT, *Enterobacter cloacae* complex; KMI, *Klebsiella michiganensis*; KPN, *Klebsiella pneumoniae*; KQI, *Klebsiella quasipneumoniae*; PRO, *Providencia rettgeri*; SER, *Serratia marcescens*.

GNB (*n* = 885) were resistant to ampicillin (95%), cefotaxime (83%) and ceftriaxone (80%), whereas they were sensitive to meropenem (13%), imipenem (15%) and tigecycline (16%) (Fig. 3a). The MICs required to inhibit the growth of 50% of organisms (that is, MIC_50_ values) of piperacillin/tazobactam, carbapenems (imipenem, meropenem and ertapenem), amikacin, fosfomycin, quinolones (ciprofloxacin and levofloxacin) and colistin were lower than their resistance breakpoints. The MIC_90_ values of all antibiotics tested were higher than their resistance breakpoints, with the exception of tigecycline, for which the MIC_90_ was lower than the epidemiological cut-off value for *Providencia* and *Proteus* species^20,21^.

Overall, GNB isolates resistant to at least one of the cephalosporins tested were less likely to cause LOS than EOS (*P* = 0.017; odds ratio (OR) = 0.63; 95% confidence interval (CI) = 0.43–0.92; Supplementary Table 3). For the statistical analysis, the outcome measurement was MFBS (deceased as response, alive as reference). Concomitant resistance to the three cephalosporins tested versus isolate susceptibility to all produced an odds ratio of 0.626; 95% CI 0.426–0.918; *P* = 0.017. In this way, concomitant resistance to the three cephalosporins tested among GNB isolates was less likely among infants who stayed alive compared to those who were deceased.

As a marker of extended-spectrum β-lactamase antibiotic resistance gene (ARG), *bla*_CTX-M-15_ was inspected. It was detected in at least nine species (*n* = 523 isolates) and found in isolates from all study sites. We also screened genomes for genes coding for carbapenem resistance (*bla*_NDM_, *bla*_OXA-48_-like variants and *bla*_KPC_). There were 146 single carbapenemase genes in ten species (*n* = 128 isolates), and two carbapenem resistance gene homologues were present in 24 isolates. *bla*_NDM-1_ (*n* = 90; Bangladesh, *n* = 23; India, *n* = 6; Nigeria, *n* = 16; Pakistan, *n* = 43; Rwanda, *n* = 1; South Africa, *n* = 1) and *bla*_NDM-5_ (*n* = 3; Bangladesh, *n* = 1; India, *n* = 1; Nigeria, *n* = 1) were mainly detected in clinical sites in South Asia, whereas *bla*_NDM-7_ was predominantly recovered from Nigeria and Pakistan (*n* = 19; Bangladesh, *n* = 1; Nigeria, *n* = 11; Pakistan, *n* = 7) (Fig. 3b). In 79 GNB, *bla*_CTX-M-15_ plus *bla*_NDM_ and/or a *bla*_OXA-48_-like variant were found. In total, 30 GNB carried a variant of the *bla*_OXA-48_-like family, with *bla*_OXA-181_ being the most frequent (Pakistan, *n* = 22; India, *n* = 1; Fig. 3b). *bla*_OXA-232_ was only found in Bangladesh (*n* = 5), and the two isolates carrying *bla*_OXA-48_ (one *K. michiganensis* and one *S. marcescens*) were from PP. *bla*_VIM_ was found in three *Pseudomonas* *aeruginosa* isolates (Bangladesh, *n* = 2 (BC, *n* = 1; BK, *n* = 1); India, *n* = 1). Both *bla*_VIM_ variants from Bangladesh were *bla*_VIM-2_, whereas the variant detected in India was *bla*_VIM-6_.

In *Enterobacter* species, *bla*_CTX-M-15_ was found in isolates recovered from both Africa and South Asia (*n* = 19; Africa, *n* = 16; South Asia, *n* = 3); however, *Enterobacter* containing carbapenemase genes (*n* = 18) were largely recovered from South Asia (South Asia, *n* = 16; Africa, *n* = 2). Five *Acinetobacter* (*A. baumannii*, *n* = 3; *Acinetobacter bereziniae*, *n* = 1; *Acinetobacter nosocomialis*, *n* = 1) were found to have *bla*_NDM-1_ (Africa, *n* = 2; South Asia, *n* = 3) (Fig. 3b). Additionally, we found *bla*_OXA-23_ within 20 *A. baumannii* isolates.

The total number of ARGs possessed by each bacterial isolate is shown in Supplementary Table 4. For each ARG increase among *E. cloacae*, we observed a 13.2% decrease in the likelihood of neonates having LOS (*P* = 0.016; 95% CI = 0.77–0.97). No other associations between ARG and onset were found (Supplementary Table 3).

Worryingly, we found that 529 (60%) of the GNB isolates tested were resistant to the first-line empirical treatment for neonatal sepsis (both ampicillin and gentamicin).

### Plasmids and carbapenemase genes

**Fig. 4:**
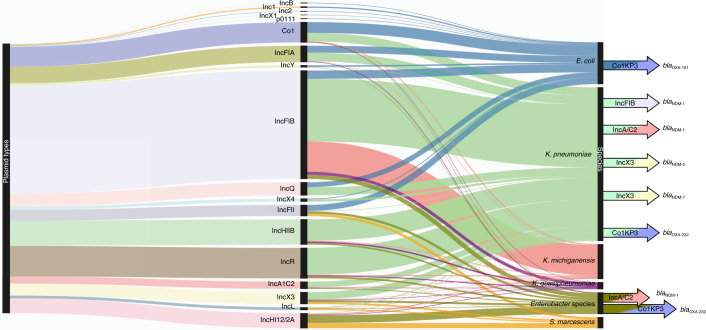
Sankey diagram showing the distribution of the different plasmid types detected linking to the GNB in which they were found. Plasmid types (left) found to carry carbapenemase AMR genes are colour coded and linked to the GNB species (right) in which the plasmid type was identified. Particular carbapenemase genes are shown on the far right.

As many ARGs are carried on mobile genetic elements such as plasmids, we searched for plasmid replicon types in isolates from the different geographical areas and, where possible, analysed linkages between plasmid type and the carriage of specific carbapenemase genes. We detected 1,124 plasmids with 45 *inc* gene variants, which we categorized into 18 plasmid groups. From these, 1,093 were found within *E. coli* (*n* = 169), *K. pneumoniae* (*n* = 623)*, K. michiganensis* (*n* = 142)*, K. quasipneumoniae* (*n* = 28)*, Enterobacter* species (*n* = 87) and *S. marcescens* (*n* = 44) (Fig. 4). There were 12 plasmid types found within the seven *S. enterica* isolates, seven among *Citrobacter* species and five among *K. variicola* isolates.

Within the six dominant plasmid carriers (Fig. 4), the most frequently detected *inc* type was IncFIB, with 255 out of 440 hits within *K. pneumoniae* genomes. *bla*_NDM-1_ was found in IncA/C2 plasmids from *K. pneumoniae* and *Enterobacter* species from PP, as well as in IncFIB plasmids from *K. pneumoniae* from India (Fig. 4 and Supplementary Fig. 10). We found IncX3 plasmids carrying *bla*_NDM-5_ in *K. pneumoniae* from NK, Nigeria, and multiple *bla*_NDM-7_ in *K. pneumoniae* and *Enterobacter* species from Nigeria and Pakistan (Fig. 4 and Supplementary Fig. 10). Col plasmid types were identified within 82 genomes. We found ColKP3 plasmids carrying *bla*_OXA-181_ or *bla*_OXA-232_ in isolates of three different species: *E. coli*, *K. pneumoniae* and *Enterobacter* species (Fig. 4 and Supplementary Fig. 10).

Our bioinformatics analysis relied on the interrogation of short-read sequencing data; therefore, it was not possible to analyse the genetic context to link carbapenemase genes and *inc* type for all genomes. Instead, a representative genome of each species/ST with the largest contig carrying the carbapenemase gene was chosen to maximize the analysis of other genetic material present, including the *inc* gene (*n* = 9; Supplementary Fig. 10). This analysis demonstrated cases where the same carbapenemase gene variant was detected in the same plasmid type across different GNB, suggesting that successful dissemination and acquisition within multiple species may be occurring. We also found cases where the same carbapenemase ARG was detected in multiple different plasmids, furthermore evidencing the spread of AMR.

### Characterization of *K. pneumoniae*

**Fig. 5:**
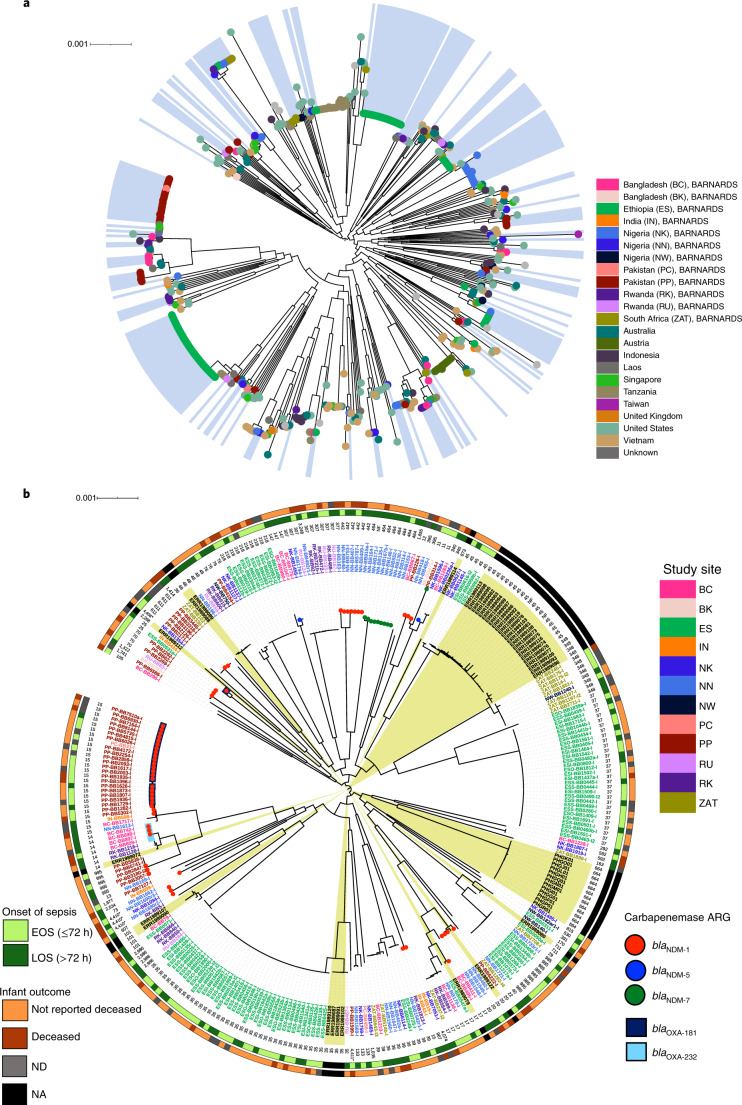
Core genome characterization of *K. pneumoniae* isolates. **a**, Five-hundred-and-fifty-nine isolates incorporating a global collection^23^. Blue shading indicates *K. pneumoniae* isolates from the BARNARDS collection. The branch labels are coloured according to country of origin. **b**, Detailed core genome characterization of 309 *K. pneumoniae* isolates (*n* = 258 BARNARDS). Yellow shading indicates isolates from other studies^15,22^ causing neonatal sepsis. The outermost rings represent infant outcome (orange) and onset of sepsis (green), followed by the ST, where asterisks represent previously unknown STs. The leaf labels are the code names (coloured according to the study site) of isolates. The branch symbols in the centre denote the carriage of carbapenemase ARGs (*bla*_NDM_ variants (circles) and *bla*_OXA-48_ group variants (squares)). NA, not applicable. Source data

*K. pneumoniae* is an important cause of neonatal sepsis in LMICs; however, there are few data analysing this species beyond antimicrobial susceptibility testing. Here, we have shown that *K. pneumoniae* was the most frequently identified GNB; therefore, the genomic diversity of this collection was scrutinized to contextualize these isolates, both within this study collection and within previously known collections^15,16,22,23^. *K. pneumoniae* (*n* = 258) was found at all clinical sites (Figs. 1 and 5, Supplementary Table 2 and Supplementary Fig. 4)—predominantly, Ethiopia (*n* = 95), Nigeria (*n* = 57) and Pakistan (*n* = 44).

Genomics analysis within both the global^15,16,22^ (Fig. 5a; see Supplementary Table 5 for literature search inclusion criteria) and neonatal sepsis context^23^ (Fig. 5b) revealed high diversity of *K. pneumoniae*, with 156 STs from 17 countries spanning five continents. BARNARDS isolates clustered with previously reported neonatal isolates, including ST45, ST48 and ST348 (refs. ^15,22,23^), but we also revealed distinct and new genetic lineages. The major AMR-related *K. pneumoniae* clades in Asia (ST11) and Europe (ST147 and ST307)^24^ were also identified in this study. We only found ST307 in Rwanda (*n* = 6) and Nigeria (*n* = 2). However, ST258, a North American clade frequently associated with *bla*_KPC_^25,26^, was absent, which accords with the absence of *bla*_KPC_ in this study. We did, however, detect one *bla*_KPC-2_ gene in a *K. quasipneumoniae* from Bangladesh (Supplementary Fig. 10). While other studies have suggested that *bla*_KPC_ *K. pneumoniae* causes neonatal sepsis, especially during nosocomial outbreaks^25^, there is currently little evidence from countries in Africa or South Asia.

BARNARDS’ *K. pneumoniae* were disseminated throughout the global phylogeny, with 57 STs (Table 1 and Fig. 5a). ST35 and ST37 were predominantly found in Ethiopia (*n* = 38/39 and *n* = 29/30, respectively). We found four ST35 *K. pneumoniae* from other neonatal sepsis publications; however, these sit on a distinct branch in the core genome phylogeny (Fig. 5b) and were more closely related to the single ST35 isolated from RU, Rwanda. ST15 isolates were largely isolated from Pakistan and all carried both *bla*_NDM-1_ and *bla*_OXA-181_ (*n* = 22/27; Fig. 5b). ST15 was almost exclusively found at the South Asian clinical sites, with a single ST15 found at NN, Nigeria. ST442 (*n* = 6) and ST464 (*n* = 8) were only found in NN and all isolates contained either *bla*_NDM-1_ (ST442) or *bla*_NDM-7_ (ST464).

Multiple different capsule types (*n* = 47 KL loci and *n* = 12 O loci) were identified in silico. ST15 isolates in Pakistan (*n* = 23) and India (*n* = 1) were all the O1v1:KL112 serotype, whereas single ST15 isolates in Bangladesh and Nigeria had different serotype combinations of O3b:KL38 and O1v1:KL48, respectively. Similarly, the ST35 isolates from Ethiopia were all O1v2:KL108, whereas *K. pneumoniae* from RU in Rwanda were O2v1:KL113. Of the eight ST348 isolates, of which seven were from South Africa and one was from NW (Nigeria), all were O1v1:KL62 and all contained the yersiniabactin virulence gene.

In total, 115 isolates had a virulence score of 1, 3 or 4, indicating the presence of yersiniabactin and/or aerobactin/salmochelin virulence genes (see the Kleborate repository^27^ and Source Data Fig. 5). The odds of LOS were 89% lower for infants with sepsis due to *K. pneumoniae* who had a virulence score of 3 or 4 compared with those with a score of 0 (*P* = 0.04; 95% CI = 0.014–0.90). Additionally, the odds of the outcome deceased were 14 times higher for infants with sepsis due to *K. pneumoniae* who had a virulence score of 3 or 4 compared with those with a virulence score of 0 (*P* = 0.001; 95% CI = 2.76–68.77; Supplementary Table 3). These results suggest that these genes may be involved in quicker onset and MFBS. Alternatively, they may reflect transmission of distinct isolates from the mother’s microbiota^6^ and from the clinical environment.

*K. pneumoniae* harboured multiple β-lactamase genes (Source Data Fig. 5)*. bla*_CTX-M-15_ was found in 220 out of 258 isolates across diverse STs and at all clinical sites, representing 42% (220/523) of total *bla*_CTX-M-15_-positive GNB. Over one-quarter (26%; 69/258) harboured a variant of *bla*_NDM_ (Figs. 4b and 5b). *bla*_NDM-1_ (*n* = 15), *bla*_NDM-5_ (*n* = 1) and *bla*_NDM-7_ (*n* = 9) were found in Nigeria (mainly NN), whereas *bla*_NDM-1_ was the dominant variant in South Asia (Fig. 4b).

All *K. pneumoniae* were resistant to ampicillin, cefotaxime, ceftriaxone and ceftazidime. *K. pneumoniae* concomitantly resistant to the three cephalosporins tested (*n* = 255) were significantly more likely to cause EOS than LOS (*P* = 0.045; OR = 0.41; 95% CI = 0.17–0.98; Supplementary Table 3). Also, 144 isolates had >15 ARGs. For each additional ARG, we observed a 5.7% (*P* = 0.028; 95% CI = 0.89–0.99) decrease in the odds of neonates having LOS compared to EOS.

Collectively, this analysis reveals a large degree of intraspecies diversity within *K. pneumoniae* pathogens causing neonatal sepsis in LMICs. While certain ST groups previously shown to be dominant in particular geographic regions were found during the BARNARDS study, we also detected several different ST groups carrying different carbapenemase ARGs and virulence determinants.

### Characterization of *E. coli*

**Fig. 6:**
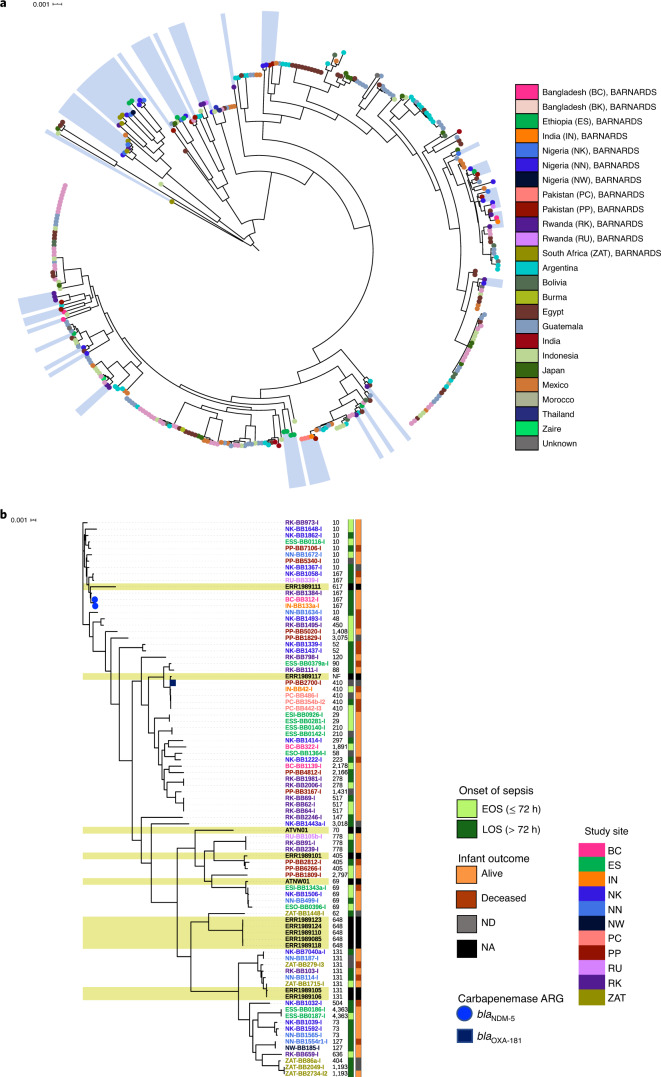
Core genome characterization of *E. coli* isolates. **a**, Three-hundred-and-sixty isolates incorporating a global collection^28^. Blue shading indicates *E. coli* isolates from the BARNARDS collection (*n* = 75). The branch labels are coloured according to country of origin. **b**, Detailed core genome characterization of 87 *E. coli* isolates (*n* = 75 BARNARDS). Yellow shading represents isolates from other studies^16,22^ causing neonatal sepsis. The colours on the right represent infant outcome (orange) and onset of sepsis (green), followed by the ST. The numbers and code names (coloured according to study site) of isolates are also given. The branch symbols denote the carriage of carbapenemase ARGs (*bla*_NDM-5_ (circles) and *bla*_OXA-181_ (squares). NF, not found. Source data

*E. coli* has previously been reported as a dominant GNB cause of neonatal sepsis across many different LMICs^18^. In light of this, we aimed to further characterize the BARNARDS *E. coli* isolates and to compare the results with existing WGS datasets (Supplementary Table 5 displays the literature search inclusion criteria). The 2014 enterotoxigenic *E. coli* collection^28^ displayed large diversity and, when analysed with the BARNARDS dataset, 90 STs across four continents and 21 countries were detected (Fig. 6a). The *E. coli* analysed herein fell within four main clades of the extended phylogeny. The greatest numbers of *E. coli* in the BARNARDS study (*n* = 75) were in Nigeria (*n* = 23; 31%) and Rwanda (*n* = 17; 23%), although *E. coli* neonatal sepsis was identified in 11 of the 12 clinical sites (excluding BK, Bangladesh). A phylogenetic analysis of BARNARDS *E. coli* revealed four main groups, each containing multiple clades (Fig. 6b), with 37 STs detected. ST10 (*n* = 9), ST131 (*n* = 6), ST410 (*n* = 5) and ST69 (*n* = 4) were the most common. In the Mentzer et al.^28^ collection, ST10 was found in five countries across Africa, Asia and South America. Of the nine ST10 *E. coli* characterized here, seven were from clinical sites within Africa (Ethiopia, *n* = 1; Nigeria, *n* = 5; Rwanda, *n* = 1) and two were from PP, Pakistan. Although all ST10 *E. coli* belonged to the same phylotype (that is, group A), each isolate had a different O:H serotype profile and the phylogenetic tree (Fig. 6b) shows variability in the branch length, indicating genomic diversity within this ST group. Generally, we found highly variable O:H serotype classification (*n* = 57 O:H combinations), irrespective of the ST or phylogroup (A–F) (Source Data Fig. 6). Of the phylotypes, A (*n* = 22), B1 (*n* = 15) and B2 (*n* = 18) were the most common.

β-lactam and aminoglycoside ARGs were most commonly found in the South Asian isolates, with the exception of *bla*_CTX-M-15_, which was also detected in *E. coli* from Africa. In total, 21 *E. coli* harboured *bla*_CTX-M-15_ and belonged to a variety of STs, including ST131, ST405, ST410, ST10 and ST167. Carbapenemase genes were detected in only three out of 75 *E. coli*. The three isolates were from South Asia, and all concomitantly carried *bla*_CTX-M-15_ plus *bla*_NDM-5_ (ST167; *n* = 2) or *bla*_OXA-181_ (ST410; *n* = 1).

No significant associations were found between phenotypic or genotypic AMR-related traits of *E. coli* and the clinical data assessed herein (Supplementary Table 3).

*E. coli* isolates were extremely diverse, suggesting that there are probably several important and worrisome lineages.

## Discussion

In the BARNARDS study, we established a methodological framework to capture and extensively characterize GNB species causing neonatal sepsis in LMICs. We isolated 916 isolates of GNB, characterized them to species level, used WGS to probe genome composition and MLST to assess intra- and interspecies diversity, and documented extremely high rates of AMR.

Most of the Gram-negative isolates from neonates with sepsis were resistant to at least one β-lactam and one aminoglycoside (597/885; 67%), as has been reported previously for cohorts in India^29^ and 26 countries in Africa^18^. World Health Organization guidelines^30^ stipulate ampicillin plus gentamicin as the first line of empirical treatment for neonatal sepsis and third-generation cephalosporins as the second line of treatment. Of note, many of the blood culture isolates from our study were resistant to both lines of treatment, meaning that treatment options are unlikely to be curative.

The identification of 58 different GNB species suggests that the aetiology of neonatal sepsis is complex. We report multiple different lineages causing infection within single species, many of which carry either resistant or putatively virulent mechanisms and several of which have previously been shown to cause neonatal sepsis (for example, ST35 and ST37 *K. pneumoniae*)^23,31^. The identification of high-risk clones, such as ST15 in *K. pneumoniae*^32^ and the global clones ST1 and ST2 in *A. baumannii*, which are notorious for nosocomial infection^33^, indicates the spread and persistence of problematic lineages in LMICs. In addition, through our comprehensive analysis, we identified 40 previously unknown STs, suggesting that well-known and previously unidentified lineages/ST groups are both co-existing and evolving.

A limitation of our study was the inability to follow up all neonates to 60 d (necessitating the exclusion of neonates who were lost to follow-up), which impacted our outcome data (Fig. 1). It is likely that additional local factors (such as the management of sepsis) contributed to mortality; therefore, we cannot attribute MFBS singularly to the presence/absence of genomic traits. Our statistical analyses were exploratory and should be interpreted as hypothesis generating only.

In summary, *Klebsiella*, *E. coli* and *Enterobacter* were the main GNB species responsible for sepsis in neonates. We report that 54% of isolated bacteria were resistant to at least one antibiotic within four to six classes of antibiotics, and observed widespread carriage of both resistance genes and virulence factors in GNB causing neonatal sepsis in LMICs. This large, observational study will inform future research into effective antimicrobial therapies for neonatal sepsis, and may underpin improved infection control practices and could be useful in the development of vaccines for neonatal sepsis in LMICs.

## Methods

### Study design and processing of blood cultures at clinical sites

A prospective cohort study was conducted through the BARNARDS network consisting of 12 clinical sites in seven countries in Africa and South Asia (Chattogram Maa-O-Shishu Hospital, Chattogram (BC) and Kumudini Women’s Medical College, Mirzapur (BK) in Bangladesh; St. Paul’s Hospital Millennium Medical College, Addis Ababa (ES) in Ethiopia; the Division of Bacteriology, ICMR-National Institute of Cholera and Enteric Diseases, Kolkata (IN) in India; National Hospital Abuja, Abuja (NN), Wuse District Hospital, Abuja (NW) and Murtala Muhammad Specialist Hospital, Kano (NK) in Nigeria; Pakistan Institute of Medical Sciences, Islamabad (PP) and Bhara Kahu Rural Health Centre, Bhara Kahu (PC) in Pakistan; University Central Hospital of Kigali, Kigali (RU) and Kabgayi Hospital, Kabgayi (RK) in Rwanda; and Tygerberg Hospital, Cape Town (ZAT) in South Africa). Ethical approval was obtained from the local ethics committee at each site before the start of the study (Supplementary Table 6). Between November 2015 and December 2017, women in labour or immediately postpartum were recruited prospectively following consent, and their neonates were followed up for the first 60 d of life (at 3, 7, 14, 28 and 60 d of life) or until study withdrawal/death. Neonates admitted to clinical sites showing signs of sepsis were also enrolled. The BARNARDS sample collection workflow is shown in Supplementary Fig. 11. Although it was not strictly a neonatal population, during this study, we employed the term neonate for all enrolments, including those between 30 and 60 d post-birth.

Documentation detailing the parameters for clinically diagnosing sepsis is available in Supplementary Fig. 1. The standard operating procedures for the laboratory processing of blood cultures and subsequent identification of bacteria, which were followed by all clinical sites (following agreement between clinical partners before the start of enrolment), are shown in Supplementary Fig. 2. Laboratory reagents (Liofilchem)—importantly, both agar media and antibiotic discs (used at the clinical sites)—were standardized throughout the network. Bacterial identification performed at each site was confirmed by WGS at Cardiff University. Antimicrobial susceptibility testing was performed twice: initially, at the local sites to guide treatment using antibiotic discs; and then at Cardiff University using the agar dilution method to establish the MICs (details below). The collected clinical data included onset of sepsis (EOS or LOS) and patient outcome following biological sepsis. Neonates that were lost to follow up were categorized along with neonates confirmed alive as ‘not reported deceased’. For the purpose of this study, EOS and LOS were defined as sepsis occurring ≤72 h and >72 h after birth, respectively. If neonates showed clinical signs of sepsis at multiple time points within the first 60 d of life, additional blood cultures were analysed. All viable bacterial species were stored on charcoal swabs (Deltalab) for transport under UN3373 regulations to Cardiff University.

At Cardiff University, GNB isolates were plated onto chromogenic urinary tract infection media supplemented with vancomycin at 10 mg l^−1^ (Liofilchem) and incubated aerobically overnight at 37 °C. Isolates were identified using a Microflex LT MALDI-TOF MS (Bruker Daltonik) with α-cyano-4-hydroxycinnamic acid matrix (Sigma–Aldrich). Bacterial isolates were stored in TS/72 beads (Technical Service Consultants) at −80 °C and the original swabs were stored at 4 °C. MICs were determined by agar dilution for a panel of 19 antibiotics and interpreted according to the EUCAST guidelines^20,21^. *E. coli* ATCC 25922 and *P. aeruginosa* ATCC 27853 strains were used as quality controls for GNB tests. Supplementary Table 7 depicts the panels of antibiotics and additional control strains used. Supplementary Table 8 defines the EUCAST interpretations used. The MIC_50_ and MIC_90_ values for each antibiotic was determined. The phenotypic metadata included the following AMR-related counts: carbapenem resistance (taken as resistance to ertapenem); methicillin resistance (taken as an oxacillin MIC > 2) and an AMR score (the number of antibiotics to which an isolate was resistant) (Supplementary Table 7).

### WGS

A single bacterial colony was transferred into 1.8 ml LB broth and incubated at 37 °C and 180 r.p.m. for 18 h. Genomic DNA was extracted using a QIAamp DNA Mini Kit (Qiagen), with an additional RNAse step, on a QIAcube (Qiagen), and quantified using a Qubit Fluorometer 3.0. Genomic libraries were prepared using a Nextera XT V2 kit (Illumina) with bead-based normalization. A total of 48 isolates were multiplexed per sequencing run to provide a depth of coverage of >15×. Paired-end WGS was performed on an Illumina MiSeq using the V3 chemistry to generate fragment lengths of up to 300 base pairs (600 cycles).

### Bioinformatics analyses

Bioinformatics analyses were performed using a high-performance computing cluster at Cardiff University (Advanced Research Computing at Cardiff (ARCCA)) and CLIMB (version 1.0)^34^. Paired-end reads (FASTQ) were subjected to quality control checks before downstream analysis. Trim Galore (version 0.4.3)^35^ was used to remove the Nextera adapter sequences and low-quality bases. Reports before and after read trimming were generated using FastQC (version 0.11.2)^36^ and collated using MultiQC (version 1.7)^37^. The mean read length and number of sequences provided on the MultiQC reports were used to determine the sequencing coverage. Paired-end reads were overlapped using Flash (version 1.2.11)^38^ and assembled into contigs using SPAdes (version 3.9.0)^39^. The trimmed FASTQ reads were mapped to the contigs using BWA (version 0.7.15)^40^ and SAMtools (version 1.3.1)^41^. Pilon (version 1.22)^42^ was used to assess any misassemblies/errors in base calling in the resulting mapped BAM file. Final genome assembly metrics were generated using QUAST (version 2.1)^43^. Bacterial species were identified using both BLAST nt (version 2.2.25; https://blast.ncbi.nlm.nih.gov/Blast.cgi; input = contigs)^44^ and PathogenWatch (version 3.13.10; https://pathogen.watch; input = contigs). MLST, virulence and plasmid genomic profiles were characterized using SRST2 (version 0.2.0)^45^ and the associated databases PlasmidFinder^46^ and VFDB^47^. Genomes were screened for ARGs using ABRicate (version 0.9.7)^48^ (databases NCBI^49^ and ResFinder^50^).

Novel alleles and novel ST profiles were submitted to BIGSdb (version 1.25.1)^51^. The O:K locus profiles for all *Klebsiella* species were determined using Kaptive (version 0.7.0)^52^ and Kleborate (version 0.2.0; https://github.com/katholt/Kleborate)^27^. The O:H serotype profiles for all *E. coli* isolates were determined using SerotypeFinder (version 2.0)^53^, and SeqSero (version 1.0)^54^ was used to determine serotypes for *Salmonella*. In silico *E. coli* phylotyping was performed using ClermonTyping (version 1.3.0)^55^. Genomes were annotated using Prokka (version 1.12)^30^. Strain relatedness analysis was performed using Roary (version 3.12.0)^56^ to create a core genome alignment and FastTree (version 2.1.11) to generate maximum likelihood phylogenetic trees. Phylogenetic trees were mid-rooted, visualized and annotated using iTOL (version 5.7)^57^. The plasmid Sankey diagram was generated using the networkD3 package in R version 3.6.2. The immediate genetic context around carbapenemase genes was performed aligning outputs from ResFinder and PlasmidFinder (in ABRicate) with Mobile Element Finder (version 1.0.1) hosted by the Center for Genomic Epidemiology (https://cge.cbs.dtu.dk/services/MobileElementFinder/). The GBK annotation file from Prokka was then analysed for image production in Geneious Prime version 2020.1.2.

### Statistical analyses

Statistical associations between clinical outcomes (onset of sepsis (EOS/LOS) and MFBS (alive/deceased)) and phenotypical and genomic traits were explored using univariable logistic regression models with the Wald test in SPSS version 26. The outcomes for the analyses regarding sepsis onset were EOS and LOS and those for MFBS were alive and deceased. Depending on whether predictor variables were continuous (AMR or ARG) or categorical (resistance versus non-resistance to ampicillin and gentamicin, concomitant resistance versus non-concomitant resistance to the three cephalosporins tested, resistance to at least one of the three cephalosporins tested versus to none, resistance versus non-resistance to ertapenem (a marker for carbapenems resistance) or *Klebsiella* species virulence scores), they were treated as covariates or factors, respectively. For species group analyses, only groups with *n* = ≥50 isolates were included, except for *B. cenocepacia* isolates, which did not carry ARGs. Statistical significance was taken at *P* ≤ 0.05, and estimated ORs are presented along with 95% CIs.

### Reporting Summary

Further information on research design is available in the Nature Research Reporting Summary linked to this article.

## Supplementary information

Supplementary Information Supplementary Figs. 1–11 and Tables 1–3 and 5–9.

Reporting Summary

Peer Review File

Supplementary Table 4 Supplementary genomics data per isolate (*n* = 916).

## Data Availability

Sequence reads have been submitted to the European Nucleotide Archive under project number PRJEB33565. Individual accession numbers and additional genomics data can be accessed in Supplementary Table 4 and the source data. The databases used for this study included VFDB (http://www.mgc.ac.cn/VFs/download.htm), NCBI (https://github.com/tseemann/abricate/tree/master/db/ncbi), ResFinder (https://github.com/tseemann/abricate/tree/master/db/resfinder), PlasmidFinder (https://bitbucket.org/genomicepidemiology/plasmidfinder/src/master), MLST (https://github.com/tseemann/mlst/tree/master/db/pubmlst), MGE (https://bitbucket.org/mhkj/mge_finder/src/master/me_finder/), SerotypeFinder (https://bitbucket.org/genomicepidemiology/serotypefinder/src/master) and SeqSero (http://www.denglab.info/SeqSero). Previously published datasets downloaded from the European Nucleotide Archive repository and used for comparative genomics analysis have the identifiers PRJEB2111, PRJEB2581 and PRJEB20875. The following genomes were downloaded from NCBI: PHGE01000000–PHGR01000000, ATNW00000000 and ATNV00000000. Source data are provided with this paper.

## Source data

Source Data Fig. 5 Raw genomics data and clinical data for 258 *K. pneumoniae* isolates used for the production of Fig. 5a,b. Source Data Fig. 6 Raw genomics data and clinical data for 75 *E. coli* isolates used for the production of Fig. 6a,b.
