# Supplementary material for: Characterization of antimicrobial-resistant Gram-negative bacteria that cause neonatal sepsis in seven low- and middle-income countries
Source: Nat Microbiol. 2021 Mar 29;6(4):512–23. doi: 10.1038/s41564-021-00870-7 (PMC8007471; doi:10.1038/s41564-021-00870-7)
Supplement: Supplementary file 1 — Supplementary Figs. 1–11 and Tables 1–3 and 5–9. [file 41564_2021_870_MOESM1_ESM.pdf]

nature portfolio

https://doi.org/10.1038/s41564-021-00870-7

Supplementary information

Characterization of antimicrobial-resistant Gram-negative bacteria that cause neonatal sepsis in seven low- and middle-income countries

In the format provided by the authors and unedited

Clinical Case Definitions for Severe Bacterial Infection (SBI) and Sepsis at Birth

<u>During the neonatal period (0-28 days):</u>
Significant **Risk Factors** at Birth:

1. PROM ≥ 18 hours
2. Maternal fever (axillary temp >37.5) at delivery time.
3. Perinatal asphyxia (APGAR ≤5 at 5 Minutes)
4. Foul smelling amniotic fluid
5. Very low birth weight (<1500g)
6. Difficult birth (dystocia)
7. Infected twin
8. Leukorrhea or untreated urinary tract infection during pregnancy.
9. Home birth

Significant **signs/symptoms: A=**very common**. B=**less common sign of severe bacterial infection/ sepsis

1. Difficulty or refusal to suckle (A)
2. Acute crying, irritability (B)
3. Drowsiness, slow reaction times, hypotonia, coma (A)
4. Bulging fontanelle (A)
5. Convulsions (A)
6. Periumbilical erythema (A)
7. Respiratory rate > 60/min (B)
8. Apnea (>15s) or bradypnea (respiratory rate <20/min) (A)
9. Hypothermia (<35.5C)/ fever (axillary temperature >37.5C) (A)
10. Purulent discharge from the eyes (A)
11. Hepatospenomegaly (B)
12. Crepitations/crackles on pulmonary auscultation (A)
13. Jaundice (B)
14. Numerous skin pustules (B)

Criteria Useable by healthcare agents→ hospitalization for medical evaluation:
1 RISK FACTOR + 1 (A) sign OR
1 RISK FACTOR + 2 (B) signs

Criteria for suspected infection after the age of 28 days (for BARNARDS up to 60 days)

1. Difficulty or refusal to suckle B
2. Acute crying/irritability B
3. Drowsiness, slow reaction time, hypotonia, coma. A
4. Bulging fontanelle A
5. Convulsions A
6. Respiratory frequency > 60 B
7. Apnoea (>15sec) or bradypnoea (respiratory rate <20) B
8. Hypothermia (<35.5.C) alternating with fever (>37.5.C) B
9. Purulent otorrhoea A
10. Purulent ocular discharge B
11. Periumbilical erythema A
12. Diarrhoea/vomiting B

Any baby with **1 category A sign** OR **2 category B signs** OR **1 category B sign +1 category A sign** will be admitted and investigated for severe infection.

Supplementary Figure S1: Neonatal Sepsis Clinical Presentation Worksheet

Appendix 2: Microbiology Standard Operating Procedure

BARNARDS

This appendix is designed to provide a standard operating procedure for the taking, storage and rectal and blood samples. Also procedures for shipment of rectal samples is provided.

Appendix M1. Sampling of Normal Flora

The mothers admitted to the birthing centre/hospital will have rectal swabs taken prior to delivery, follow the procedure detailed in '1. Procedure for collection of rectal samples'. If the baby has a normal delivery and does NOT present signs of a severe bacterial infection or sepsis (see Appendix W for definitions of Early Onset Sepsis – EOS – and Late Onset Sepsis - LOS) they will represent the control group and no further sampling is required.

If the baby stays in hospital and contracts a severe bacterial infection or sepsis or goes home and is admitted at a later date presenting with signs of sepsis (LOS) rectal samples should be taken as described in '1. Procedure for collection of rectal samples'. All results should be automatically recorded on the spreadsheet (Appendix S).

Appendix M1: Microbiology and Processing of Rectal Swabs

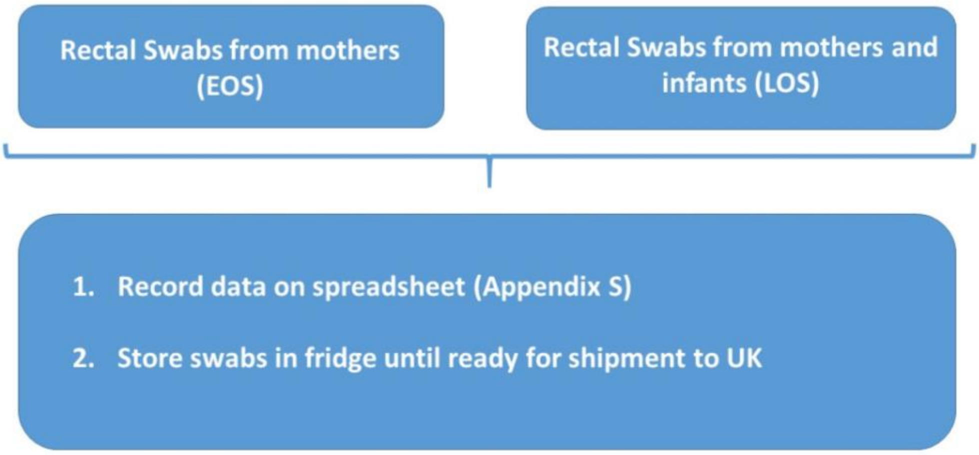

1.0

Procedure for collection of rectal samples:

1.1 Label each swab as per instructions in 'Appendix L: Labelling', in addition please add DOB and name with a waterproof marker / pen.

1.2 Moisten the swab in the appropriate transport media.

1.3 Insert swab 2.5cm into rectum and gently rotate.

1.4 Place the swab into the same tube deep enough that medium covers the cotton tips.

1.5 Seal tubes with Sellotape and place the samples in sealed, waterproof containers (i.e., plastic bags). Refrigerate at 4°C.

1.6 Shipment will be via containers UN3373 and sent using a reliable courier, organised locally.

1.7 It is important that every time a shipment of samples is sent to the UK, an electronic list of samples is emailed to miltonrl1@cardiff.ac.uk and carvalhom@cardiff.ac.uk

Appendix M2. Laboratory Confirmation of Sepsis

Infants presenting with clinical signs of sepsis from either EOS or LOS group will have blood drawn from them in an aseptic manner and specimens will be treated as described in 2.

Appendix M2: Microbiology and Processing of Blood and Blood Cultures

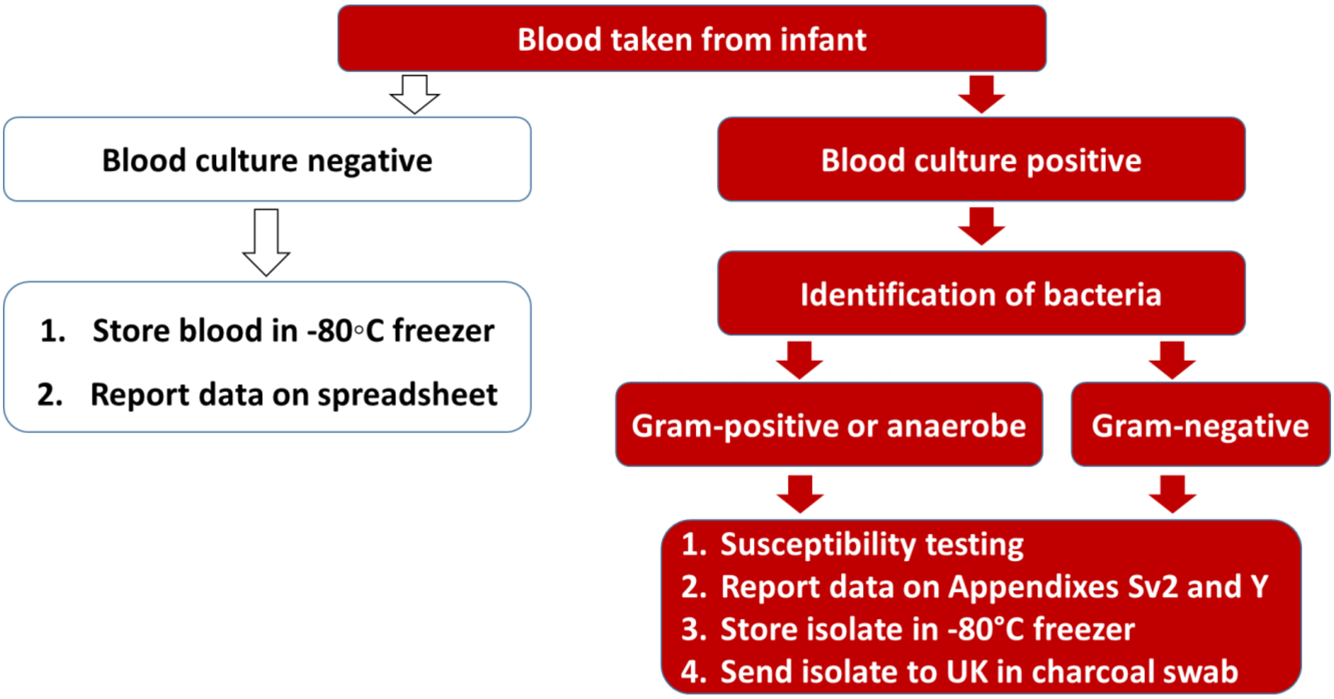

Appendix M3: Processing Blood Cultures

2.0 Procedure for collection of blood samples:
2.1 Label each blood culture bottle (BCB) using a waterproof pen or marker.
2.2 Disinfect the top of the BCB with 70% ethanol before inoculating.
2.3 Follow the in-house current procedures for drawing blood (wash hands, disinfect the skin at the venepuncture site, etc.). Avoid contamination.
2.4 Draw between 0.5ml and 2.5ml (maximum) of blood (this is dependent on the size / health of the baby) as much blood as possible should be added to the BCB.
2.5 If you can use a receptacle which separates the red blood cells then please do so, we would ideally like a 200-300µl of plasma or serum.
2.6 Incubate BCBs at 37°C for five days (minimum of four days if capacity is an issue).

3.0 Growth of bacterial cultures and isolates recovering:
3.1 Using a needle and syringe, withdraw a low volume of the positive blood culture from the BCB and lay 6 drops onto each of 3 plates of Columbia Blood Agar supplemented with 5% sterile blood (sheep or horse). Incubate plates at aerobic conditions at 35 - 37°C for 18h.
3.2 Individual pure colonies should be picked for further tests by subculture in the same media using the same conditions.

4.0 Gram-staining:
Perform Gram staining in each of the distinct isolates collected as follows:
4.1 Prepare a smear and heat gently to fix.
4.2 Flood the slide with 0.5% methyl/crystal violet and leave for 30s.
4.3 Tilt the slide, pour on sufficient (1%) Lugol's iodine to wash away the stain, cover with fresh iodine and allow to act for 30s.
4.4 Tilt the slide and wash off the iodine with 95-100% ethanol, or acetone, until colour ceases to run out of the smear.
4.5 Rinse with water.
4.6 Pour on 0.1% counterstain (neutral red, safranin or carbol fuchsin) and leave to act for about 2 minutes.

4.7 Wash with water and blot dry.
4.8 Interpretation:

Positive Result = Gram positive organisms; stain deep blue/purple.
Negative Result = Gram negative organisms; stain pink/red.

5.0 Antimicrobial susceptibility testing and detection of resistance mechanisms:

5.1 Antibiotic susceptibility testing should be performed using the Kirby-Bauer method according to EUCAST V5.0/CLSI M100-S23. Antimicrobial disks should be handled and stored according to the manufacturer's instructions.

5.2 *E. coli* ATCC25922 and *Klebsiella pneumoniae* ATCC700603 strains will be used as quality controls to monitor tests performance and should be tested alongside the isolates in study. *S. aureus* ATCC29213 will be used as an additional quality control strain to monitor vancomycin test performance for Gram-positive isolates in study.

5.3 Mueller-Hinton agar should be prepared according to the manufacturer's instructions.

5.4 Dispense the agar in Petri dishes to achieve an even depth of 4.0 mm with a maximum variation of ±0.5 mm (~25ml per plate). Store plates in sealed plastic bags at 4-8°C.

5.5 With a sterile cotton swab, suspend several colonies from fresh overnight growth in sterile saline (0.85% NaCl w/v in water) to the density of a McFarland 0.5 turbidity standard (approximately corresponding to 1–2 x 10^8 CFU/mL for *Escherichia coli*). All inoculum suspensions should optimally be used within 15 min and always within 60 min of preparation.

5.6 Dip the cotton swab into the inoculum suspension and remove the excess fluid by turning the swab against the inside of the tube.

5.7 Spread the inoculum evenly over the entire surface of the plate by swabbing in three directions or by using an automatic plate rotator.

5.8 Apply the antimicrobial disks firmly on the agar surface within 15 min of inoculation of the plates. Find the list of antibiotics to be used in List A for Gram-negative isolates. For Gram-positive isolates, additionally test cefoxitin (30µg) by disk diffusion and vancomycin by MIC strip test as described in 6.0.
(NOTE: It is important that zone diameters can be reliably measured and the maximum number of disks on a plate depends on the size of the plate, the organism and the antimicrobial agents tested. The number of disks on a plate should be limited so that unacceptable overlapping of zones is avoided. Display disks as seen in Fig.4.)

5.9 Within 15 min of application of antimicrobial disks, invert the plates and incubate at 35 ± 1°C for 16–20 h.

5.10 After incubation, a correct inoculum and satisfactorily streaked plates should result in an even confluent lawn of growth. If individual colonies can be seen, the inoculum is too light and the test should be repeated with fresh cultures.

5.11 Read plates from the back with reflected light and held above a dark background.

5.12 Measure inhibition zone diameters at the point where no obvious growth is detected by the unaided eye with a ruler, calliper or an automated zone reader.

5.13 Record the results electronically using 'Appendix S v2'.

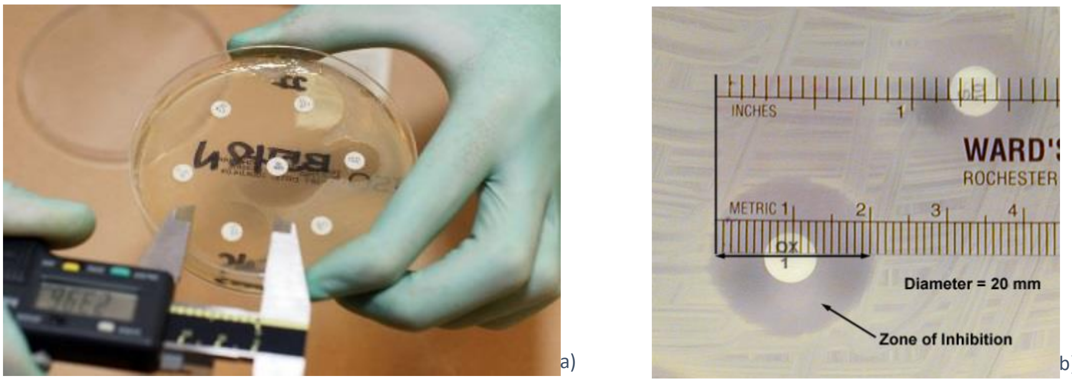

a) b)

Figure 1 – Measuring inhibition zone diameters using a) a calliper and b) a ruler.

6.0 Detection of mechanisms of resistance:

6.1 For the detection of extended spectrum beta-lactamases (ESBLs) and metallo-beta-lactamases (MBLs) production, and testing for colistin and vancomycin susceptibility, strips will be used as per manufacturer's instructions. Strips should be stored accordingly.

6.2 Quality control strains should be used as described in 3.2.

6.3 For preparation of media, bacterial cultures, bacterial suspensions and inoculation, follow steps 3.3 to 3.7.

6.4 With sterile tweezers, apply the strips as seen in Fig. 2 to the agar surface with the M.I.C. scale facing upwards and code of the strip to the outside of the plate, pressing it with the tweezers on the surface of the agar and ensure that whole length of the antibiotic gradient is in complete contact with the agar surface. Once applied, do not move the strip.

6.5 Incubate the agar plates in an inverted position at 35 ± 2°C for 16-20 hours.

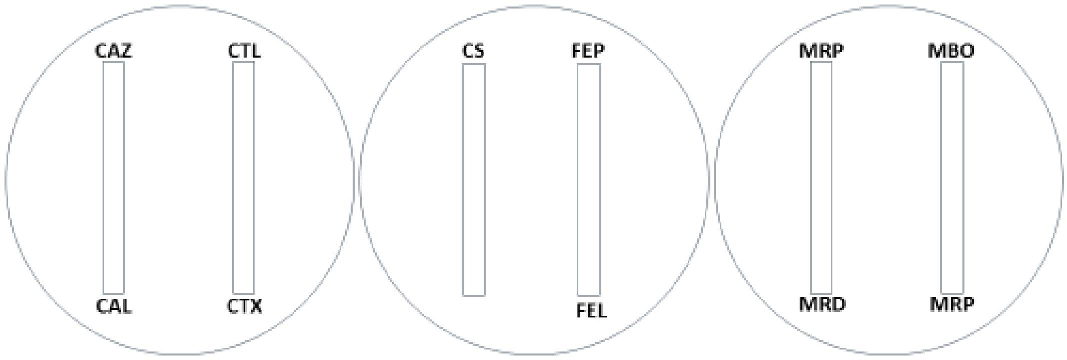

Figure 2 - Placing strips in the plate. CAZ: Ceftazidime; CAL: Ceftazidime+Clavulanic Acid; CTL: Cefotaxime+Clavulanic Acid CTX: Cefotaxime; CS: Colistin; FEP: Cefepime; FEL: Cefepime+Clavulanic Acid; MRP: Meropenem; MRD: Meropenem+EDTA; MBO: Meropenem+Boronic Acid.

7.0 Preliminary species identification of the Gram-negative isolates:

7.1 Enterosystem 18R will be used for biochemical/phenotypical identification of *Enterobacteriaceae* ONLY, as per manufacturer's instructions and exposed below.

7.2 *E. coli* ATCC25922 will be used as quality control to monitor tests performance and should be tested alongside the isolates in study.

7.3 Store the product at 2 – 8°C according to the manufacturer's instructions.

7.4 Suspend several colonies from fresh overnight growth in sterile saline (0.85% NaCl w/v in water) to the density of a McFarland 0.5[1] turbidity standard (approximately corresponding to 1–2 x 10^8 CFU/mL for *Escherichia coli*).

7.5 Take a system from its wrapper and bring it to room temperature.

7.6 Write down date and code of the microorganism.

7.7 Transfer 0.2 mL of bacterial suspension into each well of the system and overlay with 1 drop of Vaseline oil the wells 2-LDC, 3-ODC, 4-ADC, 7-UR and 8-H_2S.

7.8 Cover the system with the lid provided and incubate at 36±1°C for 12-18-24 hours.

7.9 Add 2 drops of alpha-napthol and 1 drop of NaOH 40% (ref. 80252) into the well 10-VP (VP test reagents – with the additional biochemical reagents for Enterosystem 18R). Wait for the development of a pink-red colour in about 15-20 minutes.

7.10 Add 2 or 3 drops of KOVAC'S Reagent (ref. 80252 – with the additional biochemical reagents for Enterosystem 18R) into the well 11-IND. Wait for the development of a red colour in about 1-2 minutes.

7.11 Watch for the colour change in the wells and interpret the results using table 1.

Table 1 - Results and interpretation.

Well	REACTIONS FOR THE BIOCHEMICAL IDENTIFICATION	Well color	
		**Positive reaction**	**Negative reaction**
**1-ONPG**	ONPG hydrolysis	yellow	colorless
**2-LDC**	Lysine decarboxylation	red	yellow-orange
**3-ODC**	Ornithine decarboxylation	red	yellow-orange
**4-ADC**	Arginine decarboxylation	red	yellow-orange
**5-PD**	Phenylalanine deamination	black-brown	yellow
**6-CIT**	Citrate utilization	blue-dark green	light green
**7-UR**	Urea hydrolysis	red-fuchsia	yellow-orange
**8-H_2S**	Hydrogen sulphide production	black	yellow
**9-MLN**	Malonate utilization	blue-green	yellow
**10-VP**	VP test	pink-red	yellow
**11-IND**	Indole test	red	yellow
**12-GLU**	Glucose fermentation	yellow	blu-verde
**13-MAN**	Mannitol fermentation	yellow	blu-verde
**14-INO**	Inositol fermentation	yellow	blu-verde
**15-SOR**	Sorbitol fermentation	yellow	blu-verde
**16-SAC**	Saccharose fermentation	yellow	blu-verde
**17-ARA**	Arabinose fermentation	yellow	blu-verde
**18-RAF**	Raffinose fermentation	yellow	blu-verde

7.12 Note the results on the test results form and determine the 6-digit code following instructions provided in 7.13 (NUMERICAL CODE FORMATION).

7.13 Identify the organism by using the ENTEROSYSTEM 18R Code Book (ref. 71710) or the Identification Code Disk (ref. 71711).

7.14 Numerical code formation – the biochemical tests are separated into 6 groups of 3 and a value of 1, 2 or 4 is indicated for each:

- Value 1: first test positive in each group (ONPG, ADC, UR, VP, MAN, SAC);
- Value 2: second test positive in each group (LDC, PD, H2S, IND, INO, ARA);
- Value 4: third test positive in each group (ODC, CIT, MLN, GLU, SOR, RAF);
- Value 0: every negative test.

A 6-digit code is obtained by adding together the values corresponding to positive reactions within each group. The code allows the identification of the organism under examination by using the ENTEROSYSTEM 18R Code Book (ref. 71710) or the Identification Code Disk software (ref. 71711). The example below shows how a numerical code can be formed.

7.15 Record the results electronically.

8.0 Preliminary species identification of the Gram-positive isolates:

	Group 1			Group 2			Group 3			Group 4			Group 5			Group 6		
**Test**	**ONPG**	**LDC**	**ODC**	**ADC**	**PD**	**CIT**	**UR**	**H_2S**	**MLN**	**VP**	**IND**	**GLU**	**MAN**	**INO**	**SOR**	**SAC**	**ARA**	**RAF**
**Values**	1	2	4	1	2	4	1	2	4	1	2	4	1	2	4	1	2	4
**Results**	+	+	+	–	–	–	–	–	–	–	+	+	+	–	+	–	+	–
**Sum of values**	7			0			0			6			5			2		
**CODE: 700652**	**IDENTIFICATION:** ***Escherichia coli***																	

Figure 3 - Example of numerical code formation.

8.1 Use in-house methods already established in your institution.

<u>Or, if none used,</u>

8.2 Use Staf System 18R (Liofilchem®, ref. 71630) for *Staphylococcus* sp. or Streptosystem 12R (Liofilchem®, ref. 72560) for *Streptococcus* sp. isolates according to the manufacturer's instructions.

9.0 Isolates storage:

9.1 Using a swab in charcoal transport media, take a sweep of each isolate in pure culture.

9.2 Place the swab into the same tube deep enough that medium covers the cotton tips.

9.3 Seal tubes with Sellotape and place the samples in sealed, waterproof containers (i.e., plastic bags). Refrigerate at 4°C.

9.4 Shipment will be via containers UN3373 and sent via a reliable international courier which will be organised locally.

9.5 A pure culture of each isolate should also be kept in beads for freezing at -80°C. For this a take a 10 µl loop full of the pure culture, add to the tube and mix by vortexing. Beads should be labelled with the appropriate code and in accordance with the instructions which will be supplied by Cardiff University. Each laboratory should have an appropriate database with records of all samples and isolates recovered from them.

<u>List A – Antimicrobial disks to be used and respective concentrations</u>

- Amoxicillin – 10µg
- Amoxicillin/Clavulanic acid – 20/10µg
- Piperacillin/Tazobactam – 30/6µg
- Ceftazidime – 10µg
- Cefotaxime – 5µg
- Cefepime – 30µg
- Aztreonam – 30µg
- Ertapenem – 10µg
- Imipenem – 10µg
- Meropenem – 10µg
- Trimethoprim/sulphamethoxazole – 1.25/23.75µg
- Gentamicin – 10µg
- Amikacin – 30µg
- Tigecycline – 15µg
- Fosfomycin – 200µg (CLSI_M100-S23)
- Levofloxacin – 5µg
- Ciprofloxacin – 5µg
- Nitrofurantoin – 100µg

<u>List A.1 – Additional antibiotics to be tested against Gram-positive isolates only</u>

- Vancomycin (MIC test strip)
- Cefoxitin – 30µg

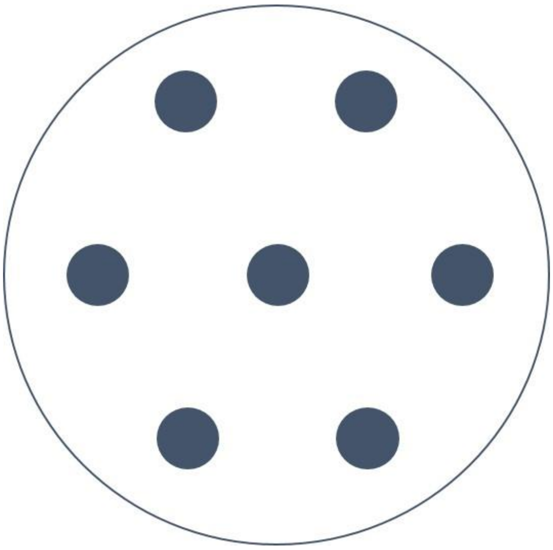

Figure 4 - Antimicrobial disks disposal. The external circle represents the Petri dish and dots represent antimicrobial disks.

Supplementary Figure S2 – Microbiology protocol used at the clinical sites during the BARNARDS study

Appendix: Enrolment consent Form

Purpose of the study

You are being invited to participate in our study focussed on the burden of antibiotic resistance in neonates from developing societies. The level of antibiotic resistance in neonatal infections and its impact on mortality in low-middle income countries is unacceptably high. This study will provide the means, support, network and tools to understand the impact of antibiotic resistance on neonatal morbidity and mortality ***(in country)*** as well as to identify possible solutions to minimise its impact.

Procedure

Once we have obtained informed consent from you, a rectal swab will be collected from you and this this swab will be sent to the UK for screening. Your swab will be assessed for multi-drug resistance (MDR) (specifically ESBL and carbapenemase positive) Gram-negative bacteria. A rectal swab will be also taken from your baby if he / she develops early ($\leq$72 hours) or late onset bacterial sepsis (>72 hours) and the results from your baby's swab will be compared to your results.

We will also require some basic demographic information and record antimicrobial therapy you were subjected to in the hospital. Our trained nurses will carry out the above mentioned procedure.

Possible risks or discomforts

There are no side effects of the study and the procedure will be discrete, quick and painless.

Possible benefits

No personal benefit, however it will improve the care and assessment of your child and provide long term strategies to improve health in ***(name country)***

Financial considerations

There is no financial compensation for your participation in this research but we'll do all laboratory tests of your sample free-of-charge.

Confidentiality

Your identity in this study will be treated as confidential. The results of the study may be published for scientific purposes but we will not give your name or include any identifiable references of you. Only the investigators of the study, data collectors will have access to your data. Your anonymity will be safeguarded under the rules we will adhere to in the Helsinki Declaration.

Termination of research study

It is important for you to understand that participation in this research is your own decision. You will decide whether you will take part in this study or not, and that you can withdraw from this study at any time during the research. This will not affect your present and future medical care in this hospital.

Available source of information

For any other problem or query please consult the local principal investigator of this study:
Name:
Email:
Telephone:
Address:

Authorisation

I have understood this consent form, and I volunteer to participate in this research study. I understand that I will receive a copy of this form. I voluntarily choose to participate, but I understand that my consent does not take away any legal rights in the case of negligence or other legal fault of anyone who is involved in this study. I further understand that nothing in this consent form is intended to replace any applicable Federal, state, or local laws.
Name of the patient: ……………………………………………………………………………
Signature: ________________________________ Date: __________________________

Supplementary Figure S3 – Enrolment and consent form for the BARNARDS study

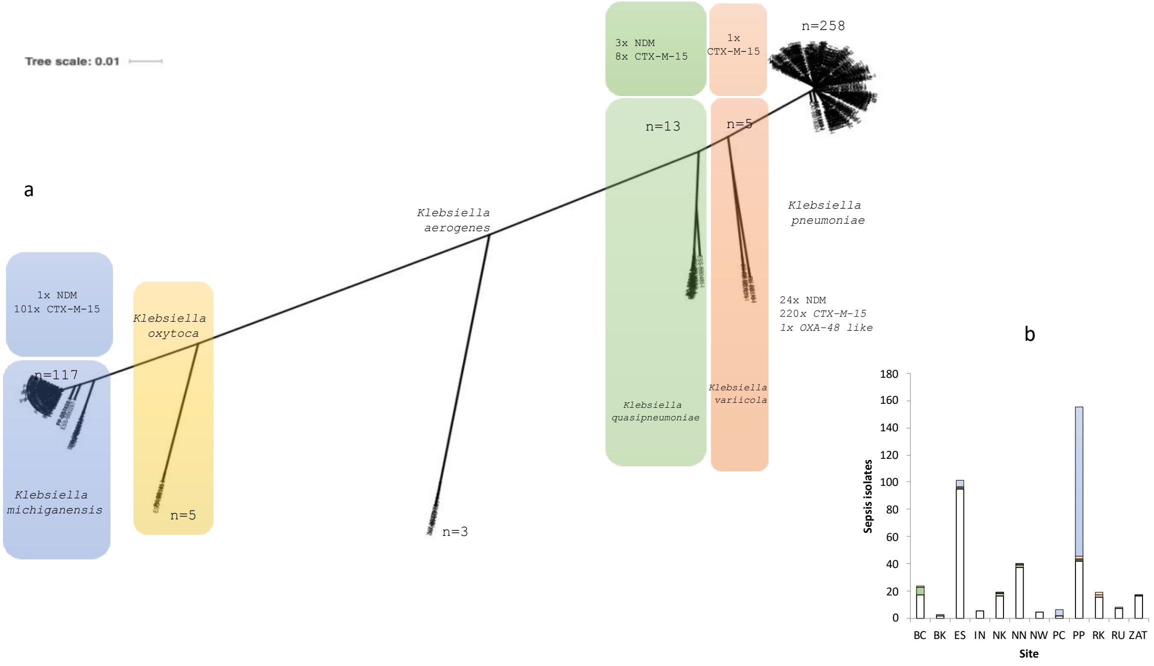

Supplementary Figure S4 – (a) Core genome phylogenetic analysis of *Klebsiella* spp. characterised during BARNARDS, using Roary (v3.12.0) and Fasttree (v2.1.11). The prevalence of *bla*$_{\text{NDM}}$, *bla*$_{\text{OXA-48}}$ group, and *bla*$_{\text{CTX-M-15}}$ per species is also shown. (b) The proportion of *Klebsiella* spp. found per site.

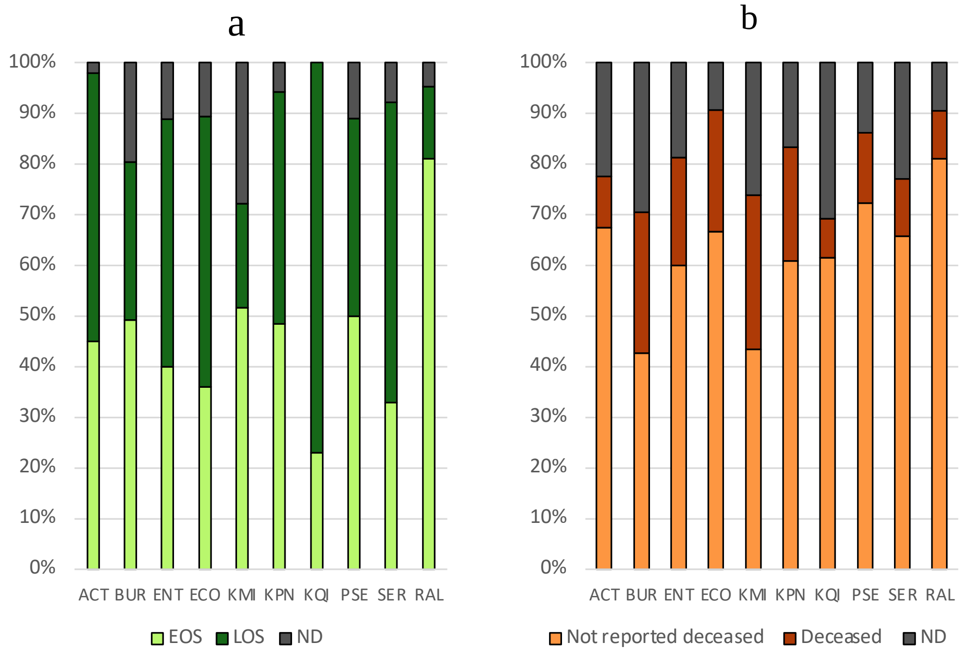

Supplementary Figure S5 - a) Onset of neonatal sepsis for the 10 most frequently identified Gram-negative (GNB) bacteria. b) Outcome of neonatal sepsis for the 10 most frequently identified GNB.

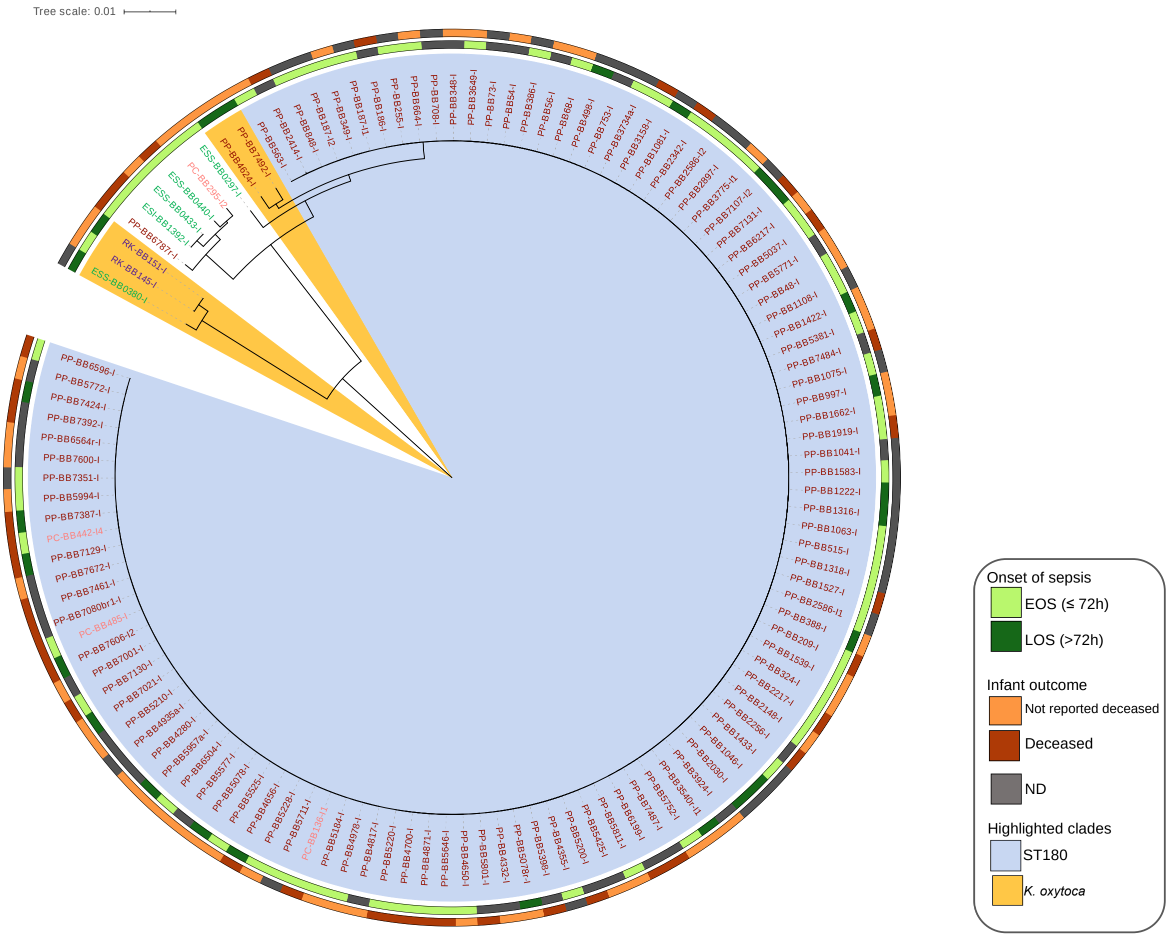

Supplementary Figure S6 - Core genome phylogenetic analysis of *Klebsiella michiganensis* species using Roary (v3.12.0) and Fasttree (v2.1.11). Leaves are colour coded per site. Closely related isolates (deciphered from identical branch lengths) are highlighted in blue and are all *K. michiganensis* from Pakistan. The orange highlighted clades denote *K. oxytoca* isolates, as per WGS identification. Onset of sepsis (green) and outcome of neonate (orange) are wrapped outside of the phylogeny.

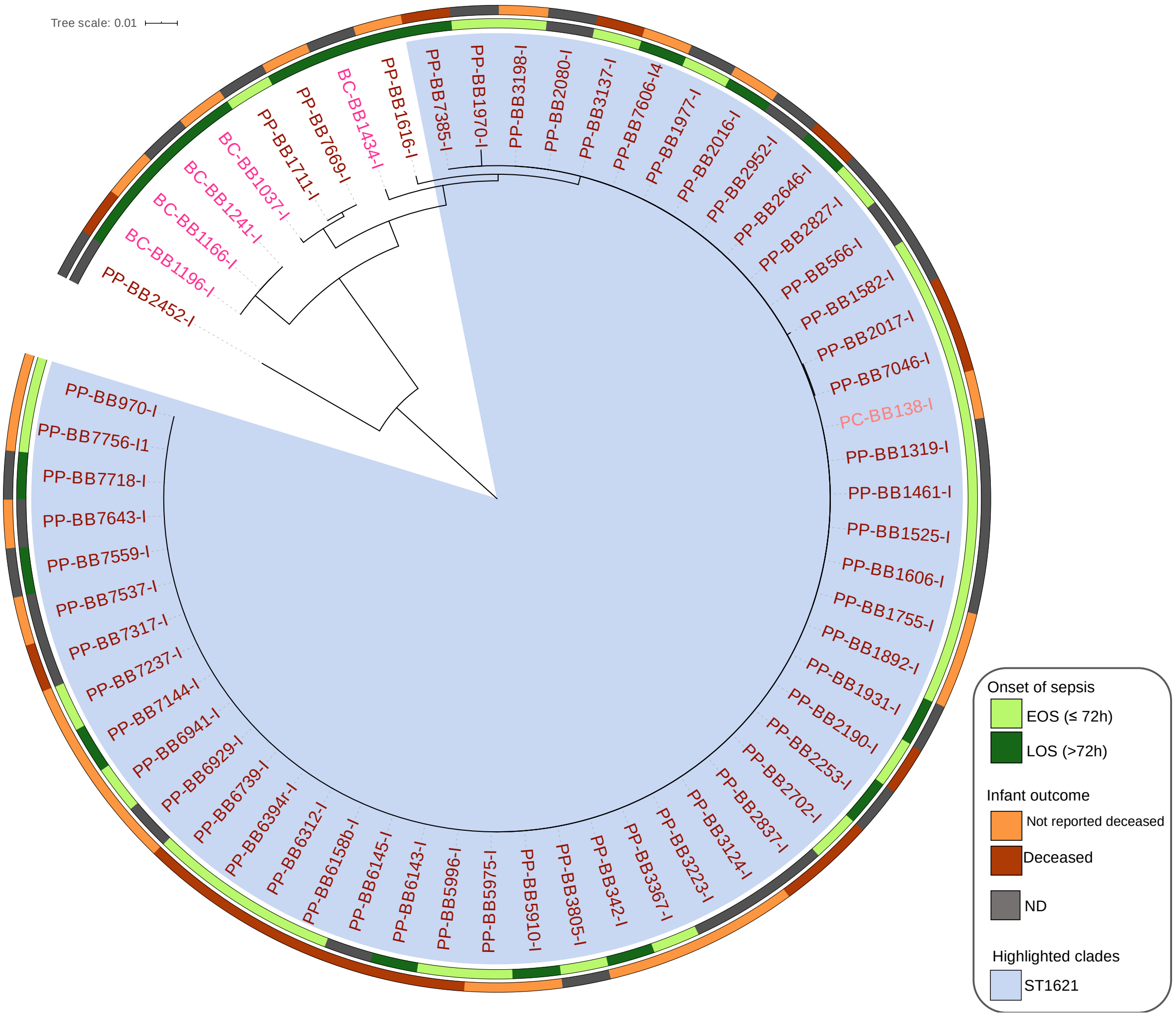

Supplementary Figure S7 - Core genome phylogenetic analysis of *Burkholderia* species using Roary (v3.12.0) and Fasttree (v2.1.11). Leaves are colour coded per site. Closely related isolates (deciphered from identical branch lengths) are highlighted in blue and are all from Pakistan. Onset of sepsis (green) and outcome of neonate (orange) are wrapped outside of the phylogeny.

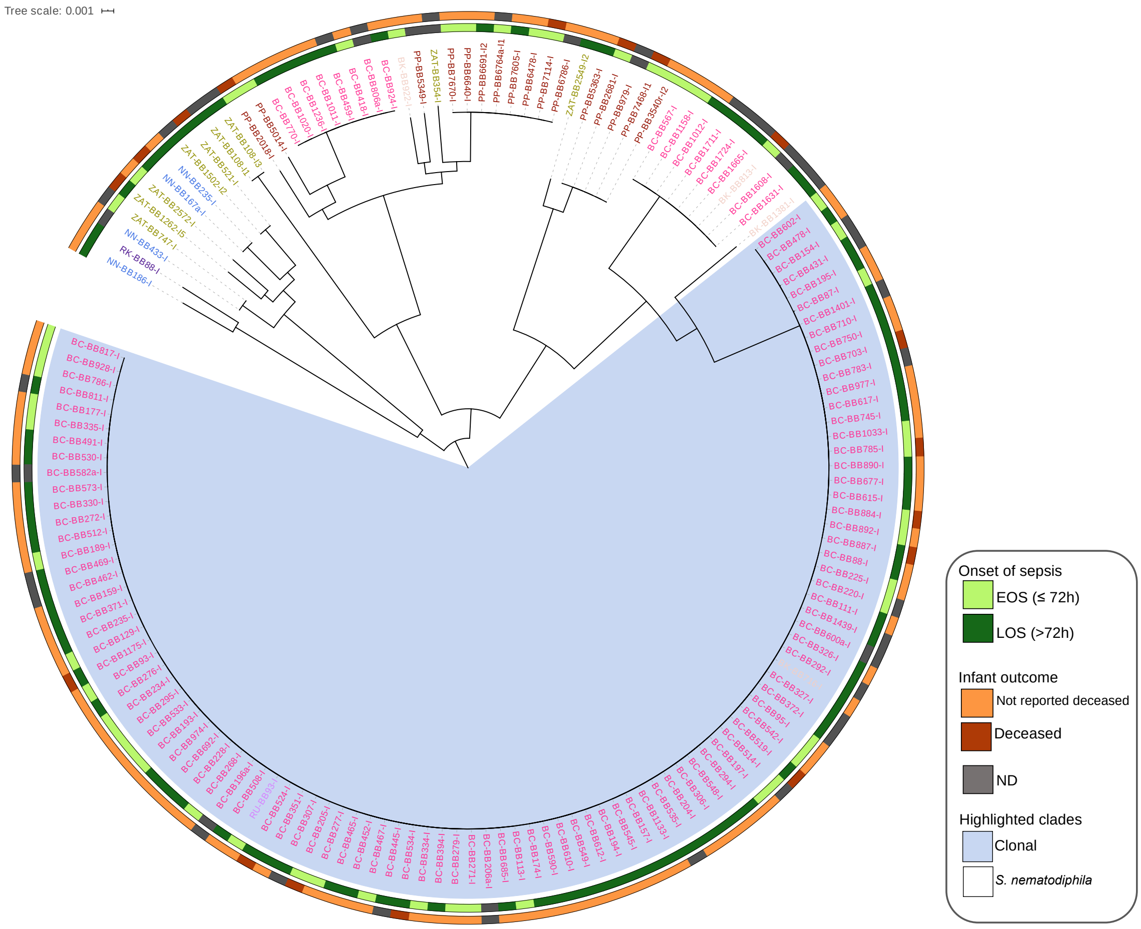

Supplementary Figure S8 - Core genome phylogenetic analysis of *Serratia* species using Roary (v3.12.0) and Fasttree (v2.1.11). Leaves are colour coded per site. Closely related isolates (deciphered from identical branch lengths) are highlighted in blue and are all *S. marcescens* from Bangladesh. The single highlighted clade (dark green) denotes a *Serratia nematopihilia*. Onset of sepsis (green) and outcome of neonate (orange) are wrapped outside of the phylogeny.

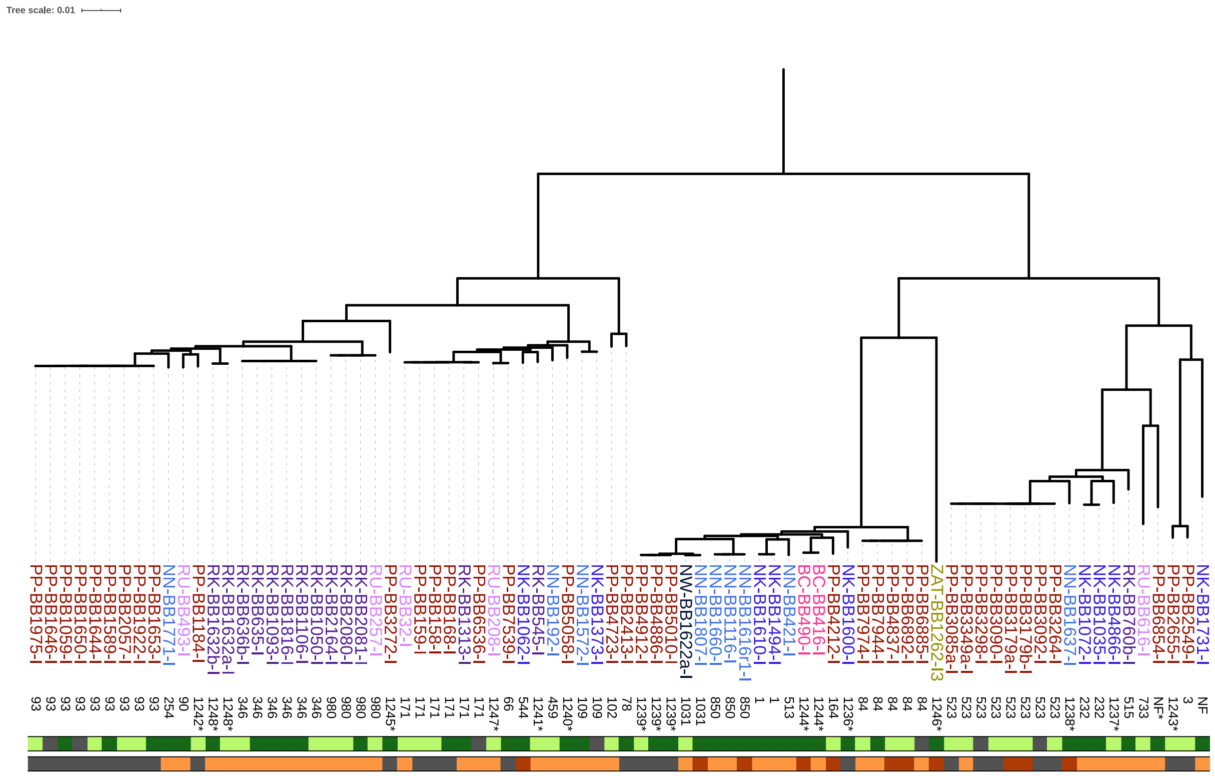

Supplementary Figure S9 - Core genome phylogenetic analysis of *Enterobacter* species using Roary (v3.12.0) and Fasttree (v2.1.11). Leaves are colour coded per site. ST is added as a text value underneath the leaf code. Onset of sepsis (green) and outcome of neonate (orange) are wrapped outside of the phylogeny.

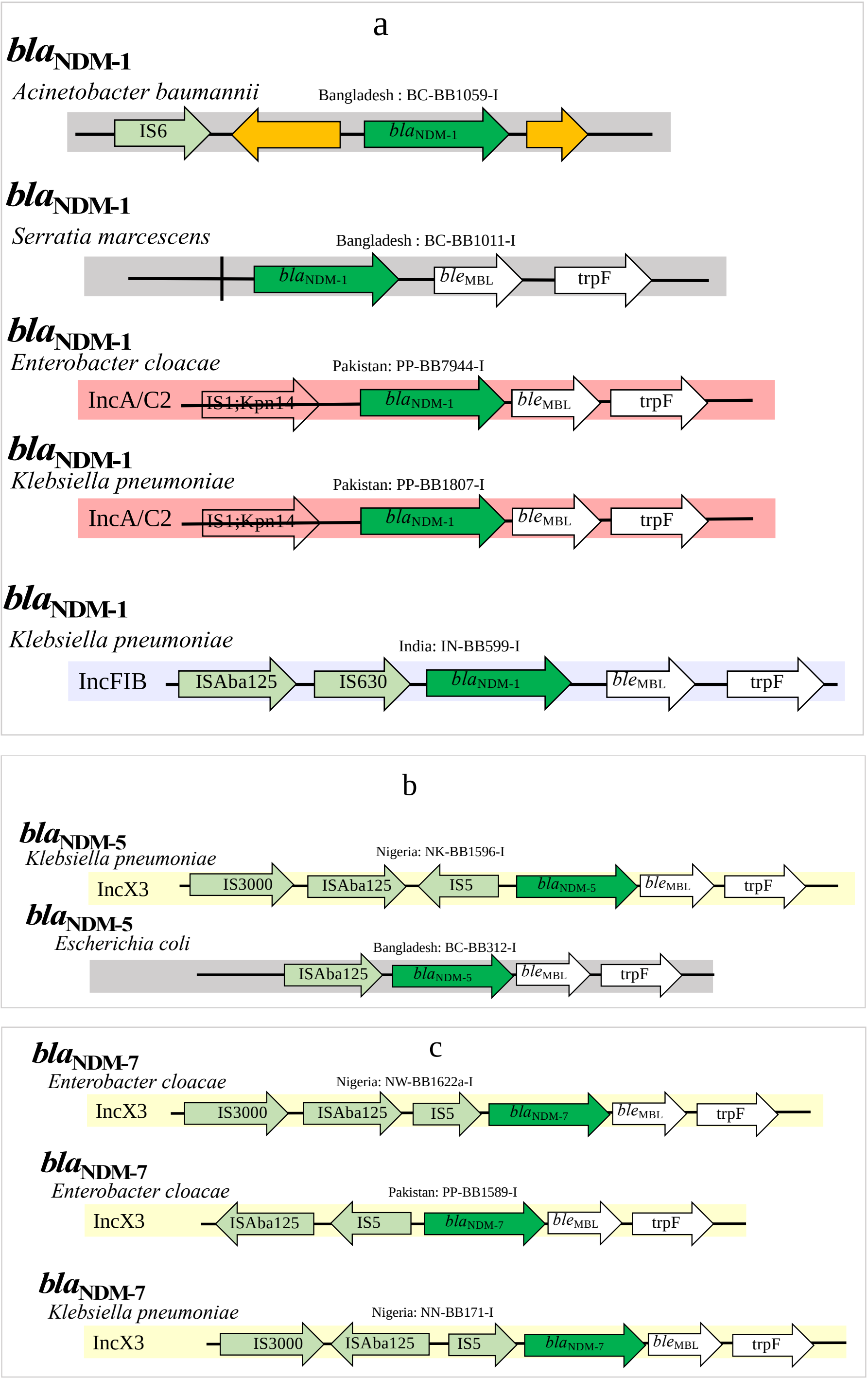

a
blaNDM-1
Acinetobacter baumannii
Bangladesh : BC-BB1059-I
IS6
blaNDM-1
blaNDM-1
Serratia marcescens
Bangladesh : BC-BB1011-I
blaNDM-1
bleMBL
trpF
blaNDM-1
Enterobacter cloacae
Pakistan: PP-BB7944-I
IncA/C2
IS1;Kpn14
blaNDM-1
bleMBL
trpF
blaNDM-1
Klebsiella pneumoniae
Pakistan: PP-BB1807-I
IncA/C2
IS1;Kpn14
blaNDM-1
bleMBL
trpF
blaNDM-1
Klebsiella pneumoniae
India: IN-BB599-I
IncFIB
ISAba125
IS630
blaNDM-1
bleMBL
trpF
b
blaNDM-5
Klebsiella pneumoniae
Nigeria: NK-BB1596-I
IncX3
IS3000
ISAba125
IS5
blaNDM-5
bleMBL
trpF
blaNDM-5
Escherichia coli
Bangladesh: BC-BB312-I
ISAba125
blaNDM-5
bleMBL
trpF
c
blaNDM-7
Enterobacter cloacae
Nigeria: NW-BB1622a-I
IncX3
IS3000
ISAba125
IS5
blaNDM-7
bleMBL
trpF
blaNDM-7
Enterobacter cloacae
Pakistan: PP-BB1589-I
IncX3
ISAba125
IS5
blaNDM-7
bleMBL
trpF
blaNDM-7
Klebsiella pneumoniae
Nigeria: NN-BB171-I
IncX3
IS3000
ISAba125
IS5
blaNDM-7
bleMBL
trpF

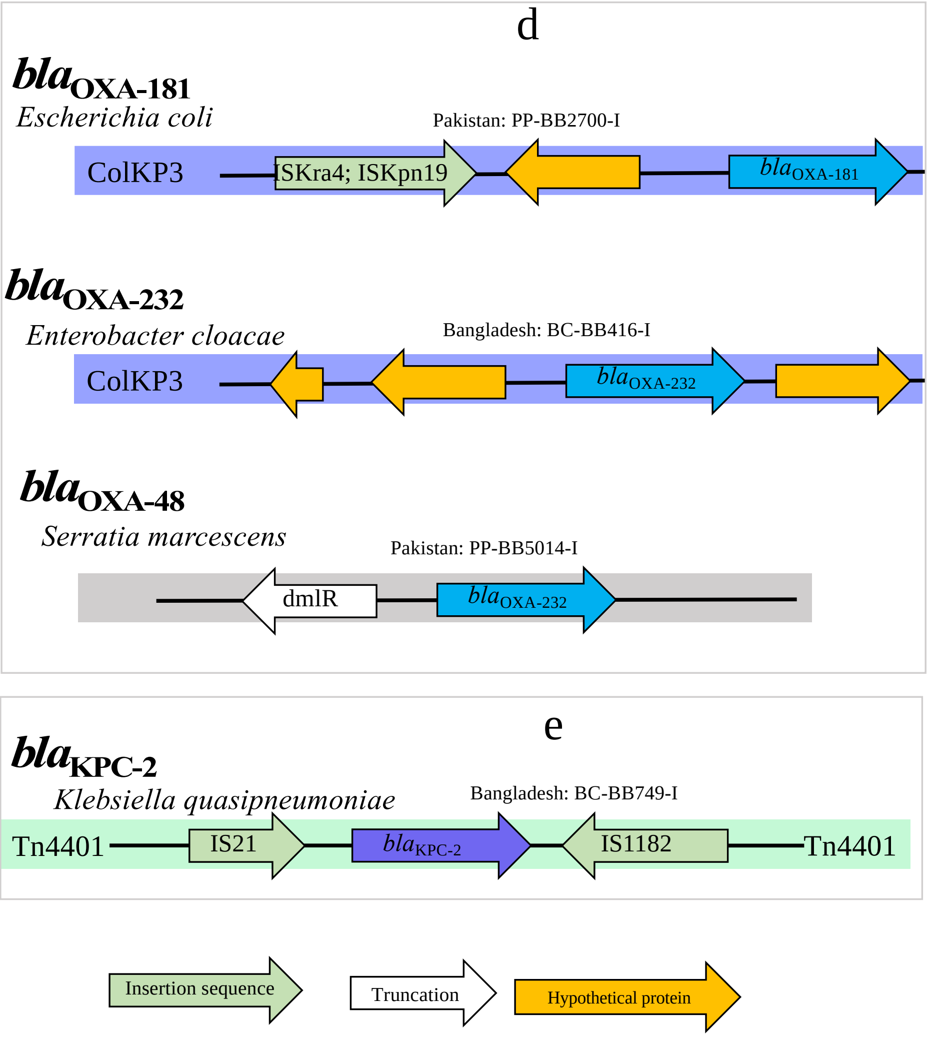

Supplementary Figure S10 – Genetic context maps of contigs containing carbapenemase genes. a) plasmid types, and insertion sequences around Gram-negative species carrying *bla*$_{NDM-1}$. b and c) plasmid types, and insertion sequences around Gram-negative species carrying *bla*$_{NDM-5}$ and *bla*$_{NDM-7}$. d) plasmid types, and insertion sequences around Gram-negative species carrying *bla*$_{OXA-48}$ like variants. e) The genetic environment around the single *bla*KPC-2 carbapenemase gene found within this study showing insertion sequences and the transposon.

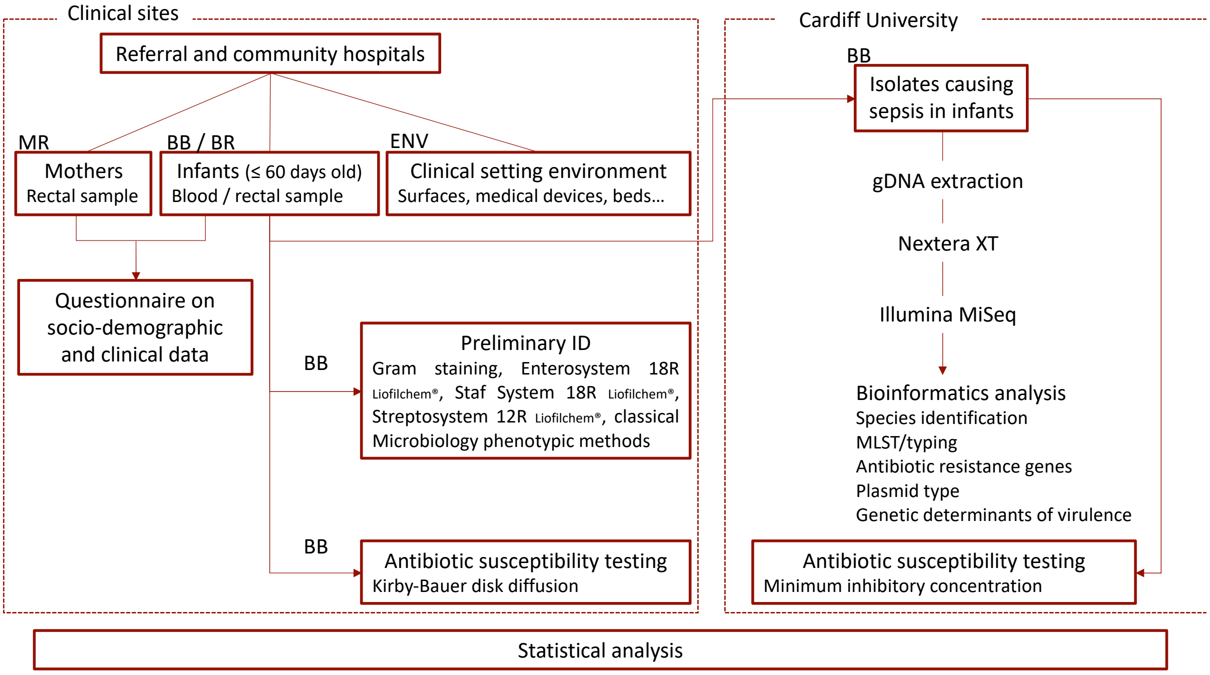

Supplementary Figure S11 - Flow diagram showing the standardised laboratory procedures and the workflow for the characterisation of BB isolates outlining the procedures at all clinical sites and at Cardiff University.

Supplementary Table 1 - Incidence of different Gram-negative bacterial species causing sepsis during BARNARDS.

	Site												Total
	BC	BK	ES	IN	NK	NN	NW	PC	PP	RK	RU	ZAT	
Achromobacter insolitus	0	0	0	0	0	2	0	0	0	0	0	0	2
Achromobacter sp.	0	0	0	0	0	0	0	0	0	1	0	0	1
Achromobacter xylosoxidans	0	0	0	1	0	1	0	0	2	0	0	0	4
Acinetobacter baumannii	7	2	7	3	3	0	0	1	8	2	2	3	38
Acinetobacter bereziniae	2	0	0	0	0	0	0	0	0	0	0	0	2
Acinetobacter junii	1	0	0	0	0	0	0	0	0	0	0	0	1
Acinetobacter nosocomialis	1	0	0	0	0	1	0	0	0	0	1	0	3
Acinetobacter radioresistens	0	0	0	0	0	0	0	0	1	0	0	0	1
Acinetobacter schindleri	0	0	0	0	2	0	0	0	0	0	0	0	2
Acinetobacter sp.	1	0	0	0	0	0	0	0	1	0	0	0	2
Aeromonas hydrophila	0	0	0	0	1	0	0	0	0	0	0	0	1
Aeromonas sp.	0	0	1	0	1	0	0	0	0	0	0	0	2
Burkholderia cenocepacia	1	0	0	0	0	0	0	1	54	0	0	0	56
Burkholderia cepacia	1	0	0	0	0	0	0	0	0	0	0	0	1
Burkholderia gladioli	0	0	0	0	0	0	0	0	1	0	0	0	1
Burkholderia sp.	3	0	0	0	0	0	0	0	0	0	0	0	3
Chryseobacterium sp.	0	0	0	0	1	0	0	0	0	0	0	0	1
Citrobacter braakii	0	0	1	0	0	0	0	0	0	0	0	0	1
Citrobacter freundii	1	1	0	0	1	0	0	0	0	0	0	0	3
Citrobacter sedlakii	0	0	0	0	0	0	0	0	0	0	1	0	1
Enterobacter asburiae	0	0	0	0	0	0	0	0	1	0	0	0	1
Enterobacter cloacae	2	0	0	0	5	9	1	0	16	10	3	0	46
Enterobacter cloacae complex	0	0	0	0	2	0	0	0	8	1	0	0	11
Enterobacter hormaechei	0	0	0	0	0	0	0	0	13	3	2	0	18
Enterobacter kobei	0	0	0	0	0	0	0	0	1	0	0	0	1
Enterobacter ludwigii	0	0	0	0	0	0	0	0	0	0	0	1	1
Enterobacter sp.	0	0	0	0	1	0	0	0	0	0	0	0	1
Enterobacter xiangfangensis	0	0	0	0	1	0	0	0	0	0	0	0	1
Escherichia coli	3	0	11	2	15	7	1	3	10	15	2	6	75
Franconibacter pulveris	0	0	0	0	1	0	0	0	0	0	0	0	1
Klebsiella aerogenes	0	0	0	0	1	1	0	0	0	0	0	1	3
Klebsiella michiganensis	0	0	4	0	0	0	0	4	109	0	0	0	117
Klebsiella oxytoca	0	0	1	0	0	0	0	0	2	2	0	0	5
Klebsiella pneumoniae	17	2	95	5	16	37	4	2	42	15	7	16	258
Klebsiella quasipneumon	6	1	1	0	2	2	0	0	1	0	0	0	13
Klebsiella variicola	1	0	0	0	0	0	0	0	1	2	1	0	5
Leclercia adecarboxylata	0	0	0	0	1	1	0	0	0	0	0	0	2
Morganella morganii	0	0	0	0	0	0	0	0	0	1	0	0	1
NA	2	0	28	1	6	2	0	7	67	3	2	1	119
Pantoea calida	0	0	0	1	0	0	0	0	0	0	0	0	1
Pantoea dispersa	1	0	0	0	0	0	0	0	0	0	0	0	1
Pantoea eucrina	0	0	0	0	0	1	0	0	0	0	0	0	1
Pantoea sp.	1	0	0	0	0	0	0	0	0	0	0	0	1
Phytobacter sp.	0	0	0	0	0	1	0	0	0	0	0	0	1
Proteus mirabilis	0	0	0	0	1	0	0	0	1	0	0	1	3
Providencia rettgeri	0	0	0	0	0	0	0	0	1	0	0	0	1
Pseudescherichia vulner	0	0	0	0	0	0	0	0	2	0	0	0	2
Pseudomonas aeruginosa	1	4	0	1	2	3	0	1	9	2	0	0	23
Pseudomonas alcaligenes	0	0	0	0	0	4	0	0	0	0	0	0	4
Pseudomonas fluorescens	1	0	0	0	0	0	0	0	0	0	0	0	1
Pseudomonas fragi	0	0	0	0	1	0	0	0	0	0	0	0	1
Pseudomonas sp.	0	0	0	0	0	0	0	0	1	0	0	0	1
Pseudomonas stutzeri	0	0	1	0	2	0	0	0	3	0	0	0	6
Ralstonia mannitolilytica	0	0	0	0	5	15	1	0	0	0	0	0	21
Raoultella ornithinolytica	0	0	0	0	0	0	0	0	2	0	0	0	2
Salmonella enterica	0	0	0	0	6	1	0	0	0	0	0	0	7
Serratia marcescens	117	3	0	0	0	4	0	0	16	1	1	9	151
Serratia nematodiphila	0	1	0	0	0	0	0	0	0	0	0	0	1
Stenotrophomonas maltophilia	0	0	0	0	0	0	0	1	2	0	0	1	4
	170	14	150	14	77	92	7	20	375	58	22	39	1038

Supplementary Table 2 - Summary of clinical, phenotypic, and genomic characterisation delineated by species/genera. n= represents the number of isolates per species with antimicrobial susceptibility profiles and whole genome sequencing data available respectively. If one n= is displayed per species, this represents both datasets for antimicrobial susceptiblity profiling and WGS. The clinical metadata includes the onset of sepsis; early onset (EOS) and late onset (LOS) and the number of MfBS. ND – not determined. Both prominent and novel STs are summarised per species. When applicable, additional in silico typing methods were performed. ARGs encoding for β-lactamases and carbapenemases are compared. The number of resistant isolates for most appropriate antibiotics (according to the species) is shown. Additionally, the most frequent plasmid incompatibility types and virulence associated genes are shown, where applicable. NA – not applicable.

Species/Genera	n=WGS/MIC	Clinical site	Onset	Outcome	Prominent STs	Novel STs	Other *in silico* typing	ARGs encoding β-lactamases	ARGs encoding carbapenemases	AMR phenotype (R)	Prominent inc plasmids	Prominent virulence determinants
Acinetobacter baumannii	n=38, n=36	Bangladesh (BC, BK), Ethiopia (ES), India (IN), Nigeria (NK), Pakistan (PC, PP), Rwanda (RK, RU), South Africa (ZAT)	16 EOS, 21 LOS, 1 ND	24 nrd, 5 deceased, 9 ND	ST1, ST2, ST575, ST1106	ST1237	N/A	*bla*$_{\text{PER-1}}$, *bla*$_{\text{TEM}}$	*bla*$_{\text{OXA-23}}$, *bla*$_{\text{OXA-51}}$, *bla*$_{\text{OXA-58}}$, *bla*$_{\text{OXA-214}}$, *bla*$_{\text{OXA-229}}$, *bla*$_{\text{OXA-235}}$, *bla*$_{\text{NDM-1}}$	IPM=20, MEM=20, AMK=20, CST=3, TGC=1	N/A	Biofilm formation, iron uptake, immune evasion
Acinetobacter spp.	n=11	Bangladesh (BC), Nigeria (NK, NN), Pakistan (PP), Rwanda (RU)	6 EOS, 7 LOS	9 nrd, 2 ND	N/A	N/A	N/A	N/A	*bla*$_{\text{OXA-23}}$, *bla*$_{\text{OXA-58}}$, *bla*$_{\text{OXA-214}}$, *bla*$_{\text{NDM-1}}$	IPM=3, MEM=3, AMK=4, CST=2, TGC=0	N/A	Biofilm formation
Burkholderia cenocepacia	n=56, n=57	Bangladesh (BC), Pakistan PC, PP)	30 EOS, 15 LOS, 11 ND	24 nrd, 16 deceased, 16 ND	ST1621	ST1621, ST1622, ST1623	N/A	N/A	N/A	CAZ=4, TZP=3, MEM=3, LVX=8	N/A	Invasion, secretion systems
Escherichia coli	n=75, n=74	Bangladesh (BC), Ethiopia (ES), India (IN), Nigeria (NK, NN, NW), Pakistan (PC, PP), Rwanda (RK, RU), South Africa (ZAT)	27 EOS, 40 LOS, 8 ND	50 nrd, 18 deceased, 7 ND	ST10, ST69, ST131, ST167, ST405, ST410	N/A	phylogroup A, B2, and B1 most common, OH serotype very diverse	*bla*$_{\text{CTX-M-15}}$, *bla*$_{\text{TEM}}$	*bla*$_{\text{OXA-181}}$, *bla*$_{\text{NDM-5}}$	GEN=15, AMP=60, CTX=29, TZP=8, CAZ=25	Col, FII, FIB	Adherence, iron uptake, protectins, toxins
Enterobacter cloacae	n=57	Bangladesh (BC), Nigeria (NK, NN, NW), Pakistan (PP), Rwanda (RK, RU)	21 EOS, 29 LOS, 7 ND	39 nrd, 13 deceased, 5 ND	ST84, ST346, ST523	ST1236, ST1238, ST1239, ST1240, ST1241, ST1243, ST1244, ST1245	N/A	*bla*$_{\text{CTX-M-15}}$, *bla*$_{\text{GES}}$, *bla*$_{\text{TEM}}$	*bla*$_{\text{OXA-232}}$, *bla*$_{\text{NDM-7}}$, *bla*$_{\text{NDM-20}}$	MEM=10, FEP=34, CAZ=40, IPM=12, CRO=40	X3, Col, FIB, HI2	N/A
Enterobacter spp.	n=23, n=22	Nigeria (NK), Pakistan (PP), Rwanda (RK, RU), South Africa (ZAT)	11 EOS, 10 LOS, 2 ND	9 nrd, 4 deceased, 10 ND	ST93, ST171	ST1237, ST1242, ST1245, ST1246, ST1248	N/A	*bla*$_{\text{CTX-M-15}}$, *bla*$_{\text{TEM}}$	*bla*$_{\text{NDM-1}}$, *bla*$_{\text{NDM-1}}$, *bla*$_{\text{NDM-7}}$	MEM=7. FEP=3, CAZ=15, IPM=7, CRO=16	X3, Col, FIB	N/A
Klebsiella michiganensis	n=117, n=116	Ethiopia (ES), Pakistan (PC, PP)	62 EOS, 21 LOS, 34 ND	49 nrd, 38 deceased, 30 ND	ST180	ST243, ST244, ST268	O1v1:KL74 serotype dominant	*bla*$_{\text{CTX-M-15}}$, *bla*$_{\text{TEM}}$	*bla*$_{\text{NDM-1}}$	CRO=110, CST=7, AMK=5, CIP=15, MEM=0	FIB	N/A
Klebsiella pneumoniae	n=258, n=254	Bangladesh (BC, BK), Ethiopia (ES), India (IN), Nigeria (NK, NN, NW), Pakistan (PC,PP)	125 EOS, 118 LOS, 15 ND	157 nrd, 58 deceased, 43 ND	ST15, ST17, ST35, ST37, ST39, ST218, ST307, ST348, ST442, ST464, ST985	ST4408, ST4410, ST4411	O1v1, O1v2, O4 dominant O-serotype. KL102, KL108, KL112, KL15 dominant KL-serotype	*bla*$_{\text{CTX-M-9}}$, *bla*$_{\text{CTX-M-15}}$, *bla*$_{\text{SHV}}$, *bla*$_{\text{TEM}}$	*bla*$_{\text{OXA-181}}$, *bla*$_{\text{OXA-232}}$, *bla*$_{\text{NDM-5}}$, *bla*$_{\text{NDM-7}}$, *bla*$_{\text{NDM-16}}$,	CRO=230, CST=7, AMK=223, CIP=140, MEM=37	FIB, FIIK, R, X3	*ybt* only.<br>*iuc and/or iro.*<br>*ybt* with *icu and/or iro.*
Klebsiella quasipneumoniae	n=13	Bangladesh (BC, BK), Ethiopia (ES), India (IN), Nigeria (NK, NN), Pakistan (PP)	3 EOS, 10 LOS	8 nrd, 1 deceased, 4 ND	ST736, ST4405	ST4405, ST4406, ST4407, ST4409	O3/O3a, O5, O12 dominant O-serotype. KL126, KL53 dominant KL-serotypes	*bla*$_{\text{CTX-M-15}}$, *bla*$_{\text{VEB-1}}$, *bla*$_{\text{SHV}}$, *bla*$_{\text{TEM}}$	*bla*$_{\text{OXA-181}}$, *bla*$_{\text{KPC-2}}$, *bla*$_{\text{NDM-1}}$, *bla*$_{\text{NDM-7}}$	CRO=10, CST=1, AMK=5, CIP=8 (plus 1 I), MEM= 2 (plus 1 I)	FIA, FIB, HI1, X3	N/A
Klebsiella variicola	n=5	Bangladesh (BC), Rwanda (RK, RU), Pakistan (PP)	4 EOS, 1 LOS	3 nrd, 1 deceased, 1 ND	All different	ST4404, ST4412, ST4413, ST4414	O2v2 dominant O-serotype. KL all different	*bla*$_{\text{CTX-M-15}}$, *bla*$_{\text{SHV}}$, *bla*$_{\text{TEM}}$	N/A	CRO=2, CST=0, AMK=0, CIP=1, MEM=0	FIB	N/A
Pseudomonas aeruginosa	n=23, n=21	Bangladesh (BC, BK), India (IN), Nigeria, (NK, NN), Pakistan (PC, PP), Rwanda (RK)	12 EOS, 8 LOS, 3 ND	18 nrd, 3 deceased, 2 ND	ST235, ST1285, ST3311	ST3311	N/A	*bla*$_{\text{VIM-1}}$, *bla*$_{\text{VEB-1}}$	N/A	MEM=3, CAZ=18, CTX=21, TZP=4, AMK=5	N/A	Adherence, antiphagocytosis, iron uptake, secretion systems, quorum sensing
Pseudomonas spp.	n=12, n=12	Bangladesh (BC), Ethiopia (ES), Nigeria (NK, NN), Pakistan (PP)	5 EOS, 6 LOS, 1 ND	9 nrd, 1 deceased, 2 ND	N/A	N/A	N/A	N/A	N/A	MEM=1, CAZ=5, CTX=9, TZP=4, AMK=1 (I)	N/A	Adherence, iron uptake, secretion systems, quorum sensing
Ralstonia mannitolilytica	n=20, n=18	Nigeria (NK, NN, NW)	16 EOS, 3 LOS, 1 ND	16 nrd, 2 deceased, 2 ND	N/A	N/A	N/A	N/A	N/A	MEM=14, TZP=10, GEN=16, AMK=16, CST=15	N/A	N/A
Salmonella enterica	n=7, n=6	Nigeria (NK, NN)	2 EOS, 7 LOS	6 nrd, 1 deceased	ST313 (*Typhimurium*)	N/A	*Typhimurium dominant serotype*	*bla*$_{\text{CTX-M-15}}$, *bla*$_{\text{TEM}}$	N/A	AMP=4, AMC=3, CRO=1, CTX=1, MEM=0	Col FIIS, FIB	N/A
Serratia marcescens	n=151, n=149	Bangladesh (BC), Nigeria (NN), Pakistan (PP), Rwanda (RK, RU), South Africa (ZAT)	50 EOS , 89 LOS, 12 ND	100 nrd, 17 deceased, 34 ND	N/A	N/A	N/A	*bla*$_{\text{CTX-M-15}}$, *bla*$_{\text{TEM}}$	*bla*$_{\text{OXA-48}}$, *bla*$_{\text{NDM-1}}$	AMK=23, CAZ=122, CTX=126, GEN=114, MEM=15	HI2, X3 - identified from isolates not within clonal [illegible]	Iron uptake
nrd= not reported deceased												

Supplementary table 3 - Estimated odds ratios from univariate logistic regression analyses using wald test. Significance was taken as p-value ≤0.05. AMR and ARG scores were treated as covariates and remaining predictor variables were treated as factors. For species groups, only groups with n≥50 isolates were included. Antibiotic resistance genes were not found among *B. cenocepacia* isolates (n=57) with our methods, thus these were not included in the analyses.

Outcome	Bacterial isolates causing sepsis	Phenotypic / genotypic predictor variables	p values	Odds ratio	95% CI (upper value)
	Overall isolates				
		Intercept	0.966	1.006	1.306
		AMR score = number of antibiotics each isolate was resistant to	0.858	1.002	1.029
	E. coli				
		Intercept	0.677	1.179	2.553
		AMR score = number of antibiotics each isolate was resistant to	0.461	1.046	1.179
	K. pneumoniae				
		Intercept	0.797	1.116	2.589
		AMR score = number of antibiotics each isolate was resistant to	0.680	0.985	1.058
	GNB isolates				
		Intercept	1.000	1.000	1.255
		Resistance to ampicillin and gentamicin	1.000	1.000	1.334
	E. coli				
		Intercept	0.338	1.304	2.245
		Resistance to ampicillin and gentamicin	0.319	1.917	6.897
	K. pneumoniae				
		Intercept	0.198	1.533	2.939
		Resistance to ampicillin and gentamicin	0.110	0.562	1.139
	GNB isolates				
		Intercept	0.237	0.875	1.092
		Concomitant resistance to the three cephalosporins tested	0.127	1.250	1.663
	E. coli				
		Intercept	0.260	1.381	2.421
		Concomitant resistance to the three cephalosporins tested	0.627	1.328	4.161
	K. pneumoniae				
		Intercept	0.079	2.125	4.924
		Concomitant resistance to the three cephalosporins tested	0.045	0.405	0.979
	GNB isolates				
		Intercept	0.029	1.481	2.105
		Resistance to at least one of the three cephalosporins tested	0.017	0.626	0.918
	E. coli				
		Intercept	0.332	1.375	2.618
		Resistance to at least one of the three cephalosporins tested	0.730	1.190	3.198
	K. pneumoniae				
		Intercept	0.232	1.833	4.957
		Resistance to at least one of the three cephalosporins tested	0.174	0.490	1.370
Onset of sepsis (LOS as response, EOS as reference category)	GNB isolates				
		Intercept	0.605	1.046	1.238
		Resistance to ertapenem, as a marker for resistance to carbapenems	0.357	0.868	1.173
	E. coli				
		Intercept	0.206	1.385	2.293
		Resistance to ertapenem, as a marker for resistance to carbapenems	0.355	2.889	27.371
	K. pneumoniae				
		Intercept	0.226	0.832	1.121
		Resistance to ertapenem, as a marker for resistance to carbapenems	0.114	1.589	2.823
	Overall isolates				
		Intercept	0.283	0.886	1.105
		ARG score = number of antibiotic resistance genes carried by each isolate	0.186	1.014	1.036
	E. cloacae				
		Intercept	0.011	4.370	13.690
		ARG score = number of antibiotic resistance genes carried by each isolate	0.016	0.868	0.974
	K. michiganensis				
		Intercept	0.007	0.378	0.765
		ARG score = number of antibiotic resistance genes carried by each isolate	0.676	0.966	1.137
	Others				
		Intercept	0.007	0.378	0.765
		ARG score = number of antibiotic resistance genes carried by each isolate	0.676	0.966	1.137
	S. marcescens	Intercept	0.634	1.257	3.222
		ARG score = number of antibiotic resistance genes carried by each isolate	0.439	1.045	1.169
	E. coli				
		Intercept	0.531	0.656	2.453
		ARG score = number of antibiotic resistance genes carried by each isolate	0.197	1.096	1.260
	K. pneumoniae				
		Intercept	0.052	2.224	4.980
		ARG score = number of antibiotic resistance genes carried by each isolate	0.028	0.943	0.994
	K. pneumoniae				
		Intercept (Kleborate virulence score = 0; none of the acquired virulence loci, i.e., negative for all of yersiniabactin, colibactin, aerobactin, salmochelin)	0.493	0.889	1.245
		Kleborate virulence score = 3 and 4 (presence of aerobactin and/or salmochelin with/without yersiniabactin (without colibactin)	0.040	0.113	0.903
		Kleborate virulence score = 1 (presence of yersiniabactin only)	0.222	1.387	2.343

Mortality following sepsis (deceased as response, alive as reference category)	Overall isolates				
		Intercept	0.000	0.372	0.507
		AMR score = number of antibiotics each isolate was resistant to	0.254	0.982	1.013
	E. coli				
		Intercept	0.005	0.281	0.689
		AMR score = number of antibiotics each isolate was resistant to	0.448	1.050	1.190
	K. pneumoniae				
		Intercept	0.022	0.310	0.842
		AMR score = number of antibiotics each isolate was resistant to	0.762	1.013	1.102
	GNB isolates				
		Intercept	0.000	0.443	0.571
		Resistance to ampicillin and gentamicin	0.007	0.627	0.883
	E. coli				
		Intercept	0.000	0.325	0.608
		Resistance to ampicillin and gentamicin	0.404	1.709	6.024
	K. pneumoniae				
		Intercept	0.061	0.500	1.031
		Resistance to ampicillin and gentamicin	0.329	0.672	1.493
	GNB isolates				
		Intercept	0.000	0.448	0.571
		Concomitant resistance to the three cephalosporins tested	0.003	0.593	0.833
	E. coli				
		Intercept	0.001	0.324	0.622
		Concomitant resistance to the three cephalosporins tested	0.471	1.542	5.000
	K. pneumoniae				
		Intercept	0.056	0.444	1.022
		Concomitant resistance to the three cephalosporins tested	0.592	0.783	1.916
	GNB isolates				
		Intercept	0.000	0.434	0.641
		Resistance to at least one of the three cephalosporins tested	0.173	0.739	1.141
	E. coli				
		Intercept	0.004	0.333	0.709
		Resistance to at least one of the three cephalosporins tested	0.711	1.227	3.621
	K. pneumoniae				
		Intercept	0.144	0.455	1.308
		Resistance to at least one of the three cephalosporins tested	0.649	0.774	2.334
	GNB isolates				
		Intercept	0.000	0.328	0.403
		Resistance to ertapenem, as a marker for resistance to carbapenems	0.592	1.104	1.585
	E. coli				
		Intercept	0.000	0.348	0.614
		Resistance to ertapenem, as a marker for resistance to carbapenems	0.497	1.917	12.529
	K. pneumoniae				
		Intercept	0.000	0.310	0.453
		Resistance to ertapenem, as a marker for resistance to carbapenems	0.166	1.577	3.005
	GPB isolates				
		Intercept	0.000	0.175	0.331
		Resistance to methicillin	0.371	1.562	4.147
	Overall isolates				
		Intercept	0.000	0.385	0.498
		ARG score = number of antibiotic resistance genes carried by each isolate	0.137	0.980	1.006
	E. cloacae				
		Intercept	0.163	0.457	1.372
		ARG score = number of antibiotic resistance genes carried by each isolate	0.509	0.962	1.078
	K. michiganensis				
		Intercept	0.289	1.464	2.965
		ARG score = number of antibiotic resistance genes carried by each isolate	0.058	0.812	1.007
	Others				
		Intercept	0.013	0.354	0.804
		ARG score = number of antibiotic resistance genes carried by each isolate	0.256	0.879	1.098
	S. marcescens				
		Intercept	0.087	0.353	1.162
		ARG score = number of antibiotic resistance genes carried by each isolate	0.204	0.907	1.054
	E. coli				
		Intercept	0.176	0.350	1.601
		ARG score = number of antibiotic resistance genes carried by each isolate	0.969	1.003	1.169
	K. pneumoniae				
		Intercept	0.303	0.614	1.553
		ARG score = number of antibiotic resistance genes carried by each isolate	0.262	0.965	1.027
	K. pneumoniae				
		Intercept (Kleborate virulence score = 0; none of the acquired virulence loci, i.e., negative for all of yersiniabactin, colibactin, aerobactin, salmochelin)	0.000	0.290	0.446
		Kleborate virulence score = 3 and 4 (presence of aerobactin and/or salmochelin with/without yersiniabactin (without colibactin)	0.001	13.778	68.765
		Kleborate virulence score = 1 (presence of yersiniabactin only)	0.454	1.278	2.429

Supplementary Table 5a – Terms used for literature search for WGS datasets of *Klebsiella pneumoniae, Escherichia coli* and *Staphylococcus aureus* isolates (n= number of hits on PubMed October 2019)

Global genomic species diversity Escherichia coli (n=28)
Global genomic species diversity Staphylococcus aureus (n=11)
Global genomic species diversity Klebsiella pneumoniae (n=10)
Escherichia coli global distribution whole genome sequencing (n=15)
Staphylococcus aureus global distribution whole genome sequencing (n=1)
Klebsiella pneumoniae global distribution whole genome sequencing (n=5)
Genomic diversity Escherichia coli (n=3527)
Genomic diversity Staphylococcus aureus (n=680)
Genomic diversity Klebsiella pneumoniae (n=266)
Whole genome sequencing surveillance Escherichia coli (n=290)
Whole genome sequencing surveillance Staphylococcus aureus (n=194)
Whole genome sequencing surveillance Klebsiella pneumoniae (n=166)

Supplementary Table 5b – Terms used for literature search for neonatal/infant sepsis studies with WGS available for *K. pneumoniae*, *E. coli* and *S. aureus* isolates (n= number of hits on PubMed August 2019)

Infant sepsis <2 months whole genome sequencing (n=27)
Klebsiella pneumoniae neonatal infant sepsis whole genome sequencing (n=2)
Escherichia coli neonatal infant sepsis whole genome sequencing (n=4)
Staphylococcus aureus neonatal infant sepsis whole genome sequencing (n=2)
neonatal sepsis genomics (n=46)
infant sepsis genomics (n=53)
blood culture positive genomics (n=56)
neonatal blood culture positive whole genome (n=3)
infant blood culture positive whole genome (n=2)
Escherichia coli sepsis whole genome sequencing (n=27)
Staphylococcus aureus sepsis whole genome sequencing (n=31)
Klebsiella pneumoniae sepsis whole genome sequencing (n=13)
Gram negative sepsis whole genome genomics (n=32)
Gram positive sepsis whole genome genomics (n=27)
Sepsis nanopore (n=6)
Sepsis PacBio (n=4)
Sepsis Illumina whole genome (n=18)
Bloodstream infection neonates genome sequencing (n=46)

Supplementary Table 6 - Ethics committees and reference numbers

Site	committees	Named PI	Reference(s)	Approval date(s)
BC	Ethical Review Committee, Bangladesh Institute of Child Health	Samir Kumar Saha	BICH-ERC-4/3/2015	15/09/2015
BK	Ethical Review Committee, Bangladesh Institute of Child Health	Samir Kumar Saha	BICH-ERC-4/3/2015	15/09/2015
ES	Boston Children's Hospital	Grace Chan	IRB-P00023058	11/08/2016
IN	Institutional Ethics Committee, National Institute of Cholera and Enteric Diseases and Institue of Post Graduate Medical Education and Research, IPGME&R Research Oversight Committee	Sulagna Basu	A-I/2016-IEC and Inst/IEC/2016/508	17/11/2016 and 04/11/2016
NK	Kano State Hospitals Management Board	Kenneth Iregbu	8/10/1437AH	13/07/2016
NN	Health Research Ethics Committee (HREC), National Hospital, Abuja	Kenneth Iregbu	NHA/EC/017/2015	27/04/2015
NW	Health Research Ethics Committee (HREC), National Hospital, Abuja	Kenneth Iregbu	NHA/EC/017/2015	27/04/2015
PC	Shaheed Zulfiqar Ali Bhutto Medical University, Pakistan Institute of Medical Sciences (PIMS) Islamabad	Rabaab Zahra	NA, signed letter from Prof. Tabish Hazir	27/05/2015
PP	Shaheed Zulfiqar Ali Bhutto Medical University, Pakistan Institute of Medical Sciences (PIMS) Islamabad	Rabaab Zahra	NA, signed letter from Prof. Tabish Hazir	27/05/2015
RK	Republic of Rwanda, National Ethics Committee	Jean-Baptiste Mazarati	No342/RNEC/2015	10/11/2015
RU	Republic of Rwanda, National Ethics Committee	Jean-Baptiste Mazarati	No342/RNEC/2015	10/11/2015
ZAT	Stellenbosch University and Tygerberg Hospital, Research projects, Western Cape Government	Shaheen Mehtar	N15/07/063	04/12/2015 and 02/02/2016

Supplementary Table 7 - panel and concentration of antibiotics tested for GNB, b) AMR phenotypic scores, c) additional quality control strains used in MIC tests.

**a) GNB**				
**Antibiotic**	**Solvent**	**Concentration (µg/ml) ranges tested**	**Additional notes**	
Amikacin	base; sterile dH2O	0, 4, 8, 16, 32		
Amoxicillin/Clavulanate	saturated NaHCO3 solution or DMSO /potassium; sterile dH2O	0, 4, 8, 16, 32	Clavulanate 2mg/L; 175µl per 35ml MHAII plate	
Ampicillin	saturated NaHCO3 solution	0, 4, 8, 16, 32		
Aztreonam	anhydrous crystalline B form; saturated NaHCO3 solution	0, 0.5, 1, 2, 4		
Cefepime	dihydrochloride; sterile dH2O	0, 0.5, 1, 2, 4		
Cefotaxime	sterile dH2O	0, 0.5, 1, 2, 4		
Ceftazidime	sterile dH2O	0, 0.5, 1, 2, 4		
Ceftriaxone	disodium; sterile dH2O	0, 0.5, 1, 2, 4		
Ciprofloxacin	0.1 HCl; sterile dH2O	0, 0.125, 0.25, 0.5, 1, 2		
Colistin	sterile dH2O	0, 1, 2, 4, 8		
Ertapenem	sterile dH2O	0, 0.25, 0.5, 1, 2		
Fosfomycin	calcium; sterile dH2O	0, 16, 32, 64, 128	12.5mg Glucose-6-phosphate (G-C-P)/ 500ml MHAII	
Gentamicin	sulphate; sterile dH2O	0, 1, 2, 4, 8		
Imipenem	monohydrate; saturated NaHCO3 solution	0, 1, 2, 4, 8		
Levofloxacin	hemihydrate; sterile dH2O	0, 0.5, 1, 2, 4		
Meropenem	trihydrate; sterile dH2O	0, 1, 2, 4, 8		
Piperacillin/Tazobactam	sterile dH2O / saturated NaHCO3 solution	0, 4, 8, 16, 32	Tazobactam 4mg/L; 175µl per 35ml MHAII plate	
Tigecycline	sterile dH2O	0, 0.5, 1, 2, 4		
Tobramycin	sulphate; sterile dH2O	0, 1, 2, 4, 8		
**b) - AMR counts**				
**Phenotypic dataset**	**Phenotypic metadata score**	**Definition**	**Score options**	**Interpretation**
a)	carbapenem resistance	resistance to meropenem	0, 1	0= susceptible, 1= resistant to meropenem
b)	ampicillin and gentamicin resistance	resistance to ampicillin and gentamicin	0, 1	0= susceptible, 1= resistance to both antibiotics
c)	3Gscore_OR	third generation cephalosporins score_OR	0, 1	0= susceptible, 1= resistance to at least one of ceftriaxone, cefotaxime or ceftazidime
d)	3Gscore	third generation cephalosporins score	0, 1	0= susceptible, 1= concomitant resistance to ceftriaxone, cefotaxime and ceftazidime
e)	AMR score	resistance to n= antibiotics	0-20	resistance to n=X antibiotics tested
**c) Additional quality control strains used in MIC tests**				
**Strain code**	**Species identification**	**Antibiotic; MIC (µg/ml)**		
A70	*Pseudomonas aeruginosa*	Fosfomycin; 16/32		
IR60	*Escherichia coli*	Tigecycline; 1		
IR35	*Klebsiella pneumoniae*	Ertapenem; >2 (highest concentration tested)		
5737	*Escherichia coli*	Cefotaxime; 4		

Supplementary Table 8) - EUCAST documents for interpretation of Minimum Inhibitory Concentration testing.

a) Breakpoints interpretation for tigecycline testing

**SPECIES**	**Document**	**S≤**	**R>**	**Comments**
Achromobacter spp.	EUCAST V9 PK-PD breakpoints	0.5	0.5	
Acinetobacter spp.	EUCAST V9 PK-PD breakpoints	0.5	0.5	
Burkholderia spp.	EUCAST V9 PK-PD breakpoints	0.5	0.5	
Citrobacter braakii	EUCAST V9 PK-PD breakpoints	0.5	0.5	
Citrobacter freundii	EUCAST tigecycline guidance doc 20181223	1	1	
Citrobacter sedlakii	EUCAST V9 PK-PD breakpoints	0.5	0.5	
Enterobacter cloacae	EUCAST tigecycline guidance doc 20181223	1	1	
Enterobacter asburiae	EUCAST tigecycline guidance doc 20181223	1	1	
Enterobacter hormaechei	EUCAST tigecycline guidance doc 20181223	1	1	
Enterobacter kobei	EUCAST tigecycline guidance doc 20181223	1	1	
Enterobacter ludwigii	EUCAST tigecycline guidance doc 20181223	1	1	
Enterobacter xiangfangensis	EUCAST V9 PK-PD breakpoints	0.5	0.5	
Franconibacter pulveris	EUCAST V9 PK-PD breakpoints	0.5	0.5	
Klebsiella pneumoniae	EUCAST tigecycline guidance doc 20181223	2	2	
Klebsiella oxytoca	EUCAST tigecycline guidance doc 20181223	1	1	
Klebsiella aerogenes	EUCAST tigecycline guidance doc 20181223	1	1	
Klebsiella michiganensis	EUCAST V9 PK-PD breakpoints	0.5	0.5	
Klebsiella quasipneumoniae	EUCAST V9 PK-PD breakpoints	0.5	0.5	
Klebsiella variicola	EUCAST V9 PK-PD breakpoints	0.5	0.5	
Morganella morganii	EUCAST tigecycline guidance doc 20181223	na	na	Isolates considered resistant
Pantoea calida	EUCAST V9 PK-PD breakpoints	0.5	0.5	
Proteus mirabilis	EUCAST V9 PK-PD breakpoints	8	8	
Providencia rettgeri	EUCAST V9 PK-PD breakpoints	4	4	
Pseudescherichia vulneris	EUCAST V9 PK-PD breakpoints	0.5	0.5	
Pseudomonas spp.	EUCAST V9 PK-PD breakpoints	0.5	0.5	
Ralstonia mannitolilytica	EUCAST V9 PK-PD breakpoints	0.5	0.5	
Raoultella ornithinolytica	EUCAST V9 PK-PD breakpoints	0.5	0.5	
Salmonella enterica	EUCAST V9 PK-PD breakpoints	0.5	0.5	
Serratia marcescens	EUCAST tigecycline guidance doc 20181223	2	2	
Serratia nematodiphila	EUCAST V9 PK-PD breakpoints	0.5	0.5	

b) - Breakpoints interpretation for GNB isolates.

**Antibiotics**	**Enterobacteriaceae**	***Pseudomonas* spp.**	***Acinetobacter* spp.**	***Stenotrophomonas maltophilia***	***Achromobacter* spp.**	***Aeromonas* spp.**	***Burkholderia* spp.**	***Ralstonia mannitolilytica***
**Ampicillin**	EUCAST V9; Enterobacterales	EUCAST V9; Enterobacterales	EUCAST V9; Enterobacterales	EUCAST V9; Enterobacterales	EUCAST V9; Enterobacterales	EUCAST V9; Enterobacterales	EUCAST V9; Enterobacterales	EUCAST V9; Enterobacterales
**Ampicillin/clavulanate**	EUCAST V9; Enterobacterales	EUCAST V9; Enterobacterales	EUCAST V9; Enterobacterales	EUCAST V9; Enterobacterales	EUCAST V9; Enterobacterales	EUCAST V9; Enterobacterales	EUCAST V9; Enterobacterales	EUCAST V9; Enterobacterales
**Piperacillin/tazobactam**	EUCAST V9; Enterobacterales	EUCAST V9; *Pseudomonas* spp.	EUCAST V9; *Pseudomonas* spp.	EUCAST V9; *Pseudomonas* spp.	EUCAST V9; Enterobacterales	EUCAST V9; Enterobacterales	EUCAST V9; *Pseudomonas* spp.	EUCAST V9; *Pseudomonas* spp.
**Ceftriaxone**	EUCAST V9; Enterobacterales	EUCAST V9; Enterobacterales	EUCAST V9; Enterobacterales	EUCAST V9; Enterobacterales	EUCAST V9; Enterobacterales	EUCAST V9; Enterobacterales	EUCAST V9; Enterobacterales	EUCAST V9; Enterobacterales
**Cefotaxime**	EUCAST V9; Enterobacterales	EUCAST V9; Enterobacterales	EUCAST V9; Enterobacterales	EUCAST V9; Enterobacterales	EUCAST V9; Enterobacterales	EUCAST V9; Enterobacterales	EUCAST V9; Enterobacterales	EUCAST V9; Enterobacterales
**Cefatzidime**	EUCAST V9; Enterobacterales	EUCAST V9; *Pseudomonas* spp.	EUCAST V9; *Pseudomonas* spp.	EUCAST V9; *Pseudomonas* spp.	EUCAST V9; Enterobacterales	EUCAST V9; *Aeromonas* spp.	EUCAST V9; *Pseudomonas* spp.	EUCAST V9; *Pseudomonas* spp.
**Cefepime**	EUCAST V9; Enterobacterales	EUCAST V9; *Pseudomonas* spp.	EUCAST V9; *Pseudomonas* spp.	EUCAST V9; *Pseudomonas* spp.	EUCAST V9; Enterobacterales	EUCAST V9; *Aeromonas* spp.	EUCAST V9; *Pseudomonas* spp.	EUCAST V9; *Pseudomonas* spp.
**Imipenem**	EUCAST V9; Enterobacterales	EUCAST V9; *Pseudomonas* spp.	EUCAST V9; *Acinetobacter* spp.	EUCAST V9; *Pseudomonas* spp.	EUCAST V9; Enterobacterales	EUCAST V9; Enterobacterales	EUCAST V9; *Pseudomonas* spp.	EUCAST V9; *Pseudomonas* spp.
**Meropenem**	EUCAST V9; Enterobacterales	EUCAST V9; *Pseudomonas* spp.	EUCAST V9; *Acinetobacter* spp.	EUCAST V9; *Pseudomonas* spp.	EUCAST V9; Enterobacterales	EUCAST V9; Enterobacterales	EUCAST V9; *Pseudomonas* spp.	EUCAST V9; *Pseudomonas* spp.
**Ertapenem**	EUCAST V9; Enterobacterales	EUCAST V9; Enterobacterales	EUCAST V9; Enterobacterales	EUCAST V9; Enterobacterales	EUCAST V9; Enterobacterales	EUCAST V9; Enterobacterales	EUCAST V9; Enterobacterales	EUCAST V9; Enterobacterales
**Aztreonam**	EUCAST V9; Enterobacterales	EUCAST V9; *Pseudomonas* spp.	EUCAST V9; *Pseudomonas* spp.	EUCAST V9; *Pseudomonas* spp.	EUCAST V9; Enterobacterales	EUCAST V9; *Aeromonas* spp.	EUCAST V9; *Pseudomonas* spp.	EUCAST V9; *Pseudomonas* spp.
**Gentamicin**	EUCAST V9; Enterobacterales	EUCAST V9; *Pseudomonas* spp.	EUCAST V9; *Acinetobacter* spp.	EUCAST V9; *Pseudomonas* spp.	EUCAST V9; Enterobacterales	EUCAST V9; Enterobacterales	EUCAST V9; *Pseudomonas* spp.	EUCAST V9; *Pseudomonas* spp.
**Amikacin**	EUCAST V9; Enterobacterales	EUCAST V9; *Pseudomonas* spp.	EUCAST V9; *Acinetobacter* spp.	EUCAST V9; *Pseudomonas* spp.	EUCAST V9; Enterobacterales	EUCAST V9; Enterobacterales	EUCAST V9; *Pseudomonas* spp.	EUCAST V9; *Pseudomonas* spp.
**Tobramycin**	EUCAST V9; Enterobacterales	EUCAST V9; Enterobacterales	EUCAST V9; *Acinetobacter* spp.	EUCAST V9; Enterobacterales	EUCAST V9; Enterobacterales	EUCAST V9; Enterobacterales	EUCAST V9; Enterobacterales	EUCAST V9; Enterobacterales
**Tigecycline**	See table a)	See table a)	See table a)	See table a)	See table a)	See table a)	See table a)	See table a)
**Minocycline**	No interpretation	No interpretation	No interpretation	No interpretation	No interpretation	No interpretation	No interpretation	No interpretation
**Fosfomycin**	EUCAST V9; Enterobacterales	EUCAST V9; Enterobacterales	EUCAST V9; Enterobacterales	EUCAST V9; Enterobacterales	EUCAST V9; Enterobacterales	EUCAST V9; Enterobacterales	EUCAST V9; Enterobacterales	EUCAST V9; Enterobacterales
**Levofloxacin**	EUCAST V9; Enterobacterales	EUCAST V9; *Pseudomonas* spp.	EUCAST V9; *Acinetobacter* spp.	EUCAST V9; *Pseudomonas* spp.	EUCAST V9; Enterobacterales	EUCAST V9; Enterobacterales	EUCAST V9; *Pseudomonas* spp.	EUCAST V9; *Pseudomonas* spp.
**Ciprofloxacin**	EUCAST V9; Enterobacterales	EUCAST V9; *Pseudomonas* spp.	EUCAST V9; *Acinetobacter* spp.	EUCAST V9; *Pseudomonas* spp.	EUCAST V9; Enterobacterales	EUCAST V9; Enterobacterales	EUCAST V9; *Pseudomonas* spp.	EUCAST V9; *Pseudomonas* spp.
**Colistin**	EUCAST V9; Enterobacterales	EUCAST V9; *Pseudomonas* spp.	EUCAST V9; *Acinetobacter* spp.	EUCAST V9; *Pseudomonas* spp.	EUCAST V9; Enterobacterales	EUCAST V9; Enterobacterales	EUCAST V9; *Pseudomonas* spp.	EUCAST V9; *Pseudomonas* spp.

Supplementary Table 9 - BARNARDS group consortium

Country	Forename	Surname	Position	Affiliation
Bangladesh	Samir	Saha	PI	Chittagong Medical College Hospital, Bangladesh
Bangladesh	Maksuda	Islam	scientist	Chittagong Medical College Hospital, Bangladesh
Bangladesh	Zabed	Bin-Ahmed	scientist	Chittagong Medical College Hospital, Bangladesh
Bangladesh	Wazir	Ahmed	clinician	Chittagong Medical College Hospital, Bangladesh
Bangladesh	Taslima	Begum	clinician	Chittagong Medical College Hospital, Bangladesh
Bangladesh	Mitu	Chowdhury	scientist	Chittagong Medical College Hospital, Bangladesh
Bangladesh	Shaila	Sharmin	scientist	Chittagong Medical College Hospital, Bangladesh
Bangladesh	Chumki	Rani Dey	Research nurse	Chittagong Medical College Hospital, Bangladesh
Bangladesh		Uttam	scientist	Chittagong Medical College Hospital, Bangladesh
Bangladesh	Abdul	Matin	clinician	Kumudini Women's Medical College, Bangladesh
Bangladesh	Sowmitra Ranjan	Chakraborty	scientist	Kumudini Women's Medical College, Bangladesh
Bangladesh	Sadia	Tasmin	clinician	Kumudini Women's Medical College, Bangladesh
Bangladesh	Dipa	Rema	Research nurse	Kumudini Women's Medical College, Bangladesh
Bangladesh	Rashida	Khatun	Research nurse	Kumudini Women's Medical College, Bangladesh
Bangladesh	Liza	Nath	Research nurse	Kumudini Women's Medical College, Bangladesh
Ethiopia	Nigatu	Balkachew	clinician	Department of Ob-Gyn, Saint Paul’s Hospital Millennium Medical College
Ethiopia	Delayehu	Bekele	Co-PI	Obstetrics and Gynaecology, Saint Paul’s Hospital Millennium Medical College
Ethiopia	Katherine	Schaughency	Data manager	Saint Paul’s Hospital Millennium Medical College
Ethiopia	Semaria	Solomon	laboratory	Department of Microbiology, Immunology and Parasitology, Saint Paul’s Hospital Millennium Medical College
Ethiopia	Zenebe	Gebreyohanes	laboratory	Department of Microbiology, Immunology and Parasitology, Saint Paul’s Hospital Millennium Medical College
Ethiopia	Rozina	Ambachew	laboratory	Department of Microbiology, Immunology and Parasitology, Saint Paul’s Hospital Millennium Medical College
Ethiopia	Oludare	Odumade	clinician	Department of Pediatrics, Boston Children’s Hospital Harvard Medical School
Ethiopia	Misgana	Haileselassie	nurse	Saint Paul’s Hospital Millennium Medical College
Ethiopia	Grace	Chan	PI	Paediatrics, Harvard Medical School
Ethiopia	Abigail	Russo	Project manager	Global Health Research Program Manager, Boston Children’s Hospital
Ethiopia	Redeat	Workneh	Project manager	Department of Pediatrics and Child Health, Saint Paul’s Hospital Millennium Medical College
Ethiopia	Gesit	Metaferia	clinician	Department of Pediatrics, Saint Paul’s Hospital Millennium Medical College
Ethiopia	Mahlet	Abayneh		Saint Paul’s Hospital Millennium Medical College
Ethiopia	Yahya Zekaria	Mohammed		Birhan for Mothers and Children Program, Saint Paul’s Hospital Millennium Medical College
Ethiopia	Tefera	Biteye		Birhan for Mothers and Children Program, Saint Paul’s Hospital Millennium Medical College
Ethiopia	Alula	Teklu	scientist	Saint Paul’s Hospital Millennium Medical College
Ethiopia	Wendimagegn	Gezahegn	clinician	Department of Pediatrics, Saint Paul’s Hospital Millennium Medical College
India	Partha Sarathi	Chakravorty	clinician	Obstetrics & Gynecology, IPGMER & SSKM Hospital

India	Anuradha	Mukherjee	clinician	Division of Bacteriology, ICMR-National Institute of Cholera and Enteric Diseases,Kolkata-700010,India
India	Ranjan Kumar	Nandy	Co-PI	Scientist-F, Division of Bacteriology, ICMR-National Institute of Cholera and Enteric Diseases
India	Samarpan	Roy	Data entry operator	ICMR-National Institute of Cholera and Enteric Diseases
India	Anuradha	Sinha	Deputy manager	R&D, Zydus Cadila
India	Sharmi	Naha	laboratory	ICMR-SRF, Division of Bacteriology, ICMR-National Institute of Cholera and Enteric Diseases
India	Sukla Saha	Malakar	laboratory	ICMR-National Institute of Cholera and Enteric Diseases
India	Siddhartha	Bose	laboratory	Medical Laboratory Technologist, Swasthya Bhawan
India	Monaki	Majhi	laboratory	Baruipur SD Hospital
India	Subhasree	Sahoo	nurse	
India	Putul	Mukherjee	nurse	
India	Sumitra Kumari	Routa	nurse	
India	Chaitali	Nandi	nurse	
India	Sulagna	Basu	PI	Scientist F, Division of BacteriologyICMR-National Institute of Cholera and Enteric Diseases,
India	Bijan	Saha	scientist	Neonatology, IPGMER & SSKM Hospital
India	Pinaki	Chattopadhyay	scientist	Neonatal Microbiology, IPGMER & SSKM Hospital
Nigeria	Fatima Zara Isa	Modibbo	clinician	54gene
Nigeria	Stella	Uwaezuoke	clinician	Federal Medical Centre Jabi, Abuja, Nigeria
Nigeria	Dilichukwu	Meduekwe	clinician	Wuse District Hospital, Nigeria
Nigeria	Khairiyya	Muhammad	laboratory	National Hospital Abuja, Nigeria
Nigeria	Queen	Nsude	laboratory	Wuse District Hospital, Nigeria
Nigeria	Ifeoma	Ukeh	laboratory	National Hospital Abuja, Nigeria
Nigeria	Mary-Joe	Okenu	laboratory	National Hospital Abuja, Nigeria
Nigeria	Akpulu	Chinenye	laboratory	54gene
Nigeria	Samuel	Yakubu	laboratory	National Hospital Abuja, Nigeria
Nigeria	Vivian	Asunugwo	laboratory	Wuse District Hospital, Nigeria
Nigeria	Folake	Aina	NYS Corp Member	National Hospital Abuja, Nigeria
Nigeria	Isibong	Issy	NYS Corp Member	National Hospital Abuja, Nigeria
Nigeria	Dolapo	Adekeye	NYS Corp Member	National Hospital Abuja, Nigeria
Nigeria	Adiele	Eunice	nurse	Wuse District Hospital, Nigeria
Nigeria	Abdulmlik	Amina	nurse	Wuse District Hospital, Nigeria
Nigeria	R	Oyewole	nurse	National Hospital Abuja, Nigeria
Nigeria	I	Oloton	nurse	National Hospital Abuja, Nigeria
Nigeria	BC	Nnaji	nurse	National Hospital Abuja, Nigeria
Nigeria	M	Umejiego	nurse	National Hospital Abuja, Nigeria
Nigeria	PN	Anoke	nurse	National Hospital Abuja, Nigeria
Nigeria	S	Adebayo	nurse	National Hospital Abuja, Nigeria
Nigeria	GO	Abegunrin	nurse	National Hospital Abuja, Nigeria

Nigeria	OB	Omotosho	nurse	National Hospital Abuja, Nigeria
Nigeria	R	Ibrahim	nurse	National Hospital Abuja, Nigeria
Nigeria	B	Igwe	nurse	National Hospital Abuja, Nigeria
Nigeria	M	Abroko	nurse	National Hospital Abuja, Nigeria
Nigeria	K	Balami	nurse	National Hospital Abuja, Nigeria
Nigeria	L	Bayem	nurse	National Hospital Abuja, Nigeria
Nigeria	C	Anyanwu	nurse	National Hospital Abuja, Nigeria
Nigeria	H	Haruna	nurse	National Hospital Abuja, Nigeria
Nigeria	J	Okike	nurse	National Hospital Abuja, Nigeria
Nigeria	K	Goroh	nurse	National Hospital Abuja, Nigeria
Nigeria	M	Boi-Sunday	nurse	National Hospital Abuja, Nigeria
Nigeria	Augusta	Ugafor	nurse	National Hospital Abuja, Nigeria
Nigeria	Maryam	Makama	nurse	National Hospital Abuja, Nigeria
Nigeria	Kaniba	Ndukwe	nurse	National Hospital Abuja, Nigeria
Nigeria	Anastesia	Odama	nurse	National Hospital Abuja, Nigeria
Nigeria	Hadiza	Yusuf	nurse	National Hospital Abuja, Nigeria
Nigeria	Patience	Wachukwu	nurse	National Hospital Abuja, Nigeria
Nigeria	Kachalla	Yahaya	nurse	National Hospital Abuja, Nigeria
Nigeria	Titus	Kalade Colsons	nurse	National Hospital Abuja, Nigeria
Nigeria	Mercy	Kura	nurse	National Hospital Abuja, Nigeria
Nigeria	Damilola	Orebiyi	nurse	National Hospital Abuja, Nigeria
Nigeria	Kenneth C.	Iregbu	PI	National Hospital Abuja, Nigeria
Nigeria	Chukwuemeka	Mmadueke	clinician	Wuse District Hospital, Nigeria
Nigeria	Lamidi	Audu	clinician	National Hospital Abuja, Nigeria
Nigeria	Nura	Idris	clinician	Director Kano State Health Trust Fund
Nigeria	Safiya	Gambo	clinician	Murtala Muhammad Specialist Hospital, Nigeria
Nigeria	Jamila	Ibrahim	clinician	Department of paediatric Muhammad Abdullahi Wase Teaching Hospital
Nigeria	Edwin	Precious	scientist	Department of Microbiology Aminu Kano Teaching Hospital
Nigeria	Ashiru	Hassan		Murtala Muhammad Specialist Hospital, Nigeria
Nigeria	Shamsudden	Gwadabe	scientist	Department of Physiology NorthWest University Kano
Nigeria	Adeola	Adeleye Falola	scientist	54gene
Nigeria	Muhammad	Aliyu	scientist	Murtala Muhammad Specialist Hospital, Nigeria
Nigeria	Amina	Ibrahim	scientist	Murtala Muhammad Specialist Hospital, Nigeria
Nigeria	Aisha Sani	Mukaddas	scientist	Murtala Muhammad Specialist Hospital, Nigeria
Nigeria	Rashida Yakubu	Khalid	scientist	Murtala Muhammad Specialist Hospital, Nigeria
Nigeria	Fatima Ibrahim	Alkali	scientist	Murtala Muhammad Specialist Hospital, Nigeria

Nigeria	Maryam Yahaya	Muhammad	scientist	Murtala Muhammad Specialist Hospital, Nigeria
Nigeria	Fatima Mohammad	Tukur	scientist	Murtala Muhammad Specialist Hospital, Nigeria
Nigeria	Surayya Mustapha	Muhammad	scientist	Department of Biotechnology Federal University Dutse
Nigeria	Adeola	Shittu	scientist	Murtala Muhammad Specialist Hospital, Nigeria
Nigeria	Murjanatu	Bello	scientist	Murtala Muhammad Specialist Hospital, Nigeria
Nigeria	Muhammad Abubakar	Hassan	scientist	Murtala Muhammad Specialist Hospital, Nigeria
Nigeria	Fatima Habib	Sa ad	scientist	Murtala Muhammad Specialist Hospital, Nigeria
Nigeria	Aishatu	Kassim	scientist	Murtala Muhammad Specialist Hospital, Nigeria
Pakistan	Haider	Shirazi	Co-PI	Neonatology Department, Pakistan Institute of Medical Sciences
Pakistan	Adil	Muhammad	laboratory	Department of Microbiology, Quaid-i-Azam University, Islamabad 45320, Pakistan
Pakistan	Rabaab	Zahra	PI	Department of Microbiology, Quaid-i-Azam University, Islamabad 45320, Pakistan
Pakistan	Syed Najeeb	Ullah	scientist	Department of Microbiology, Quaid-i-Azam University, Islamabad 45320, Pakistan
Pakistan	Muhammad	Hilal Jan	project manager	Department of Microbiology, Quaid-i-Azam University, Islamabad 45320, Pakistan
Pakistan	Rubina	Kamran	clinician	Pakistan Institute of Medical Sciences
Pakistan		Sajana	research nurse	Pakistan Institute of Medical Sciences
Pakistan	Jazba	Saeed	research nurse	Pakistan Institute of Medical Sciences
Pakistan	Noreen	Maqsood	research nurse	Pakistan Institute of Medical Sciences
Pakistan	Maria	Zafar	research nurse	Pakistan Institute of Medical Sciences
Pakistan	Saraeen	Sadiq	research nurse	Pakistan Institute of Medical Sciences
Pakistan	Sumble	Ahsan	research nurse	Pakistan Institute of Medical Sciences
Pakistan	Madiha	Tariq	scientist	Pakistan Institute of Medical Sciences
Pakistan	Sidra	Sajid	scientist	Pakistan Institute of Medical Sciences
Pakistan	Hasma	Mustafa	scientist	Pakistan Institute of Medical Sciences
Pakistan	Anees-ur	Rehman	local project manager	Pakistan Institute of Medical Sciences
Pakistan	Atif	Muhammad	local project manager	Pakistan Institute of Medical Sciences
Pakistan	Gahssan	Mehmood	local project manager	Pakistan Institute of Medical Sciences
Pakistan	Mahnoor	Nisar	local project manager	Pakistan Institute of Medical Sciences
Pakistan	Shermeen	Akif	local project manager	Pakistan Institute of Medical Sciences
Pakistan	Tahira	Yasmeen	local project manager	Pakistan Institute of Medical Sciences
Pakistan	Sabir	Nawaz	community coordinator	Pakistan Institute of Medical Sciences
Pakistan	Anam Shanal	Atta	community coordinator	Pakistan Institute of Medical Sciences
Pakistan	Mian	Laiq-ur-Rehman	community coordinator	Pakistan Institute of Medical Sciences

Pakistan	Robina	Kousar	community nurse	Pakistan Institute of Medical Sciences
Pakistan	Kalsoom	Bibi	community nurse	Pakistan Institute of Medical Sciences
Pakistan	Kosar	Waheed	community nurse	Pakistan Institute of Medical Sciences
Pakistan	Zainab	Majeed	community nurse	Pakistan Institute of Medical Sciences
Pakistan	Ayesha	Jalil	data support	Pakistan Institute of Medical Sciences
Rwanda	Espoir	Kajibwami	clinician	Kabgayi Hospital, Kabgayi, Gitarama, Rwanda
Rwanda	Aniceth	Rucogoza	laboratory	Microbiology Laboratory Director, National Reference Laboratory Rwanda.
Rwanda	Innocent	Nzabahimana	laboratory	The University Teaching Hostpial (CHUK), Kigali, Rwanda
Rwanda	Mazarati	Jean-Baptiste	PI	Head of Biomedical Services National Reference Laboratory, Kigali Rwanda
Rwanda	Lucie	Gaju	project manager	The University Teaching Hostpial (CHUK), Rwanda
Rwanda	Kankundiye	Riziki	nurse/scientist	The University Teaching Hostpial (CHUK), Kigali, Rwanda
Rwanda	Brigette	Uwamahoro	nurse/scientist	The University Teaching Hostpial (CHUK), Kigali, Rwanda
Rwanda	Rachel	Uwera	nurse/scientist	The University Teaching Hostpial (CHUK), Kigali, Rwanda
Rwanda	Eugenie	Nyiratuza	nurse/scientist	The University Teaching Hostpial (CHUK), Kigali, Rwanda
Rwanda	Kumwami	Muzungu	nurse/scientist	The University Teaching Hostpial (CHUK), Kigali, Rwanda
Rwanda	Violette	Uwitonze	nurse/scientist	Kabgayi Hospital, Kabgayi, Gitarama, Rwanda
Rwanda	Marie C	Horanimpundu	nurse/scientist	Kabgayi Hospital, Kabgayi, Gitarama, Rwanda
Rwanda	Francine	Nzeyimana	nurse/scientist	Kabgayi Hospital, Kabgayi, Gitarama, Rwanda
Rwanda	Prince	Mitima	nurse/scientist	Kabgayi Hospital, Kabgayi, Gitarama, Rwanda
South Africa	Angela	Dramowski	clinician	Department of Paediatrics and child health, Stellenbosch University, Stellenbosch, Cape Town
South Africa	Andrew	Whitelaw	Co-PI	Division of Medical Microbiology, Stellenbosch University, Tygerberg Hospital, Cape Town
South Africa	Lauren	Paterson	laboratory	Division of Medical Microbiology, Stellenbosch University, Tygerberg Hospital, Cape Town
South Africa	Mary	Frans	nurse	Tygerberg Hospital, Cape Town
South Africa	Marvina	Johnson	nurse	Tygerberg Hospital, Cape Town
South Africa	Eveline	Swanepoel	nurse	Tygerberg Hospital, Cape Town
South Africa	Zoleka	Bojana	nurse	Tygerberg Hospital, Cape Town
South Africa	Mieme	du Preez	nurse	Tygerberg Hospital, Cape Town
South Africa	Shaheen	Mehtar	PI	Faculty of Medicine and Health Sciences, Unit for Infection Prevention and Control, Stellenbosch University
South Africa	Andre	Bulabula	scientist	Faculty of Medicine and Health Sciences, Unit for Infection Prevention and Control, Stellenbosch University
The Netherlands	Feiyan	Liu	scientist	Leiden Academic Centre for Drug Research, Leiden University, Leiden, The Netherlands
The Netherlands	Johan GC	van Hasselt	scientist	Leiden Academic Centre for Drug Research, Leiden University, Leiden, The Netherlands
UK	Timothy	Walsh	PI	Institute of Infection and Immunity, School of Medicine, Cardiff University
UK	Kirsty	Sands	scientist	Institute of Infection and Immunity, School of Medicine, Cardiff University
UK	Maria	Carvalho	scientist	Institute of Infection and Immunity, School of Medicine, Cardiff University
UK	Rebecca	Milton	project manager	Institute of Infection and Immunity, School of Medicine, Cardiff University
UK	Kathryn	Thomson	scientist	Institute of Infection and Immunity, School of Medicine, Cardiff University

UK	Edward	Portal	scientist	Institute of Infection and Immunity, School of Medicine, Cardiff University
UK	Jordan	Mathias	scientist	Institute of Infection and Immunity, School of Medicine, Cardiff University
UK	Calie	Dyer	Data manager	Institute of Infection and Immunity, School of Medicine, Cardiff University
UK	Ana	Ferreira	scientist	Institute of Infection and Immunity, School of Medicine, Cardiff University
UK	Robert	Andrews	scientist	Institute of Infection and Immunity, School of Medicine, Cardiff University
UK	John	Watkins	scientist	Institute of Infection and Immunity, School of Medicine, Cardiff University
UK	David	Gillespie	scientist	Centre for Trials Research, Cardiff University
UK	Kerry	Hood	Co-PI	Centre for Trials Research, Cardiff University
UK	Katie	Taiyai	scientist	Centre for Trials Research, Cardiff University
UK	Nigel	Kirby	scientist	Centre for Trials Research, Cardiff University
UK	Maria	Nieto	scientist	Institute of Infection and Immunity, School of Medicine, Cardiff University
UK	Thomas	Hender	scientist	Institute of Infection and Immunity, School of Medicine, Cardiff University
UK	Patrick	Hogan	scientist	Institute of Infection and Immunity, School of Medicine, Cardiff University
UK	Habiba	Saif	scientist	Institute of Infection and Immunity, School of Medicine, Cardiff University
UK	Brekhna	Hassan	scientist	Institute of Infection and Immunity, School of Medicine, Cardiff University
UK	Ellis	Jones	scientist	Institute of Infection and Immunity, School of Medicine, Cardiff University
UK	Matthew	Barrell	scientist	Institute of Infection and Immunity, School of Medicine, Cardiff University
UK	Ian	Boostrom	scientist	Institute of Infection and Immunity, School of Medicine, Cardiff University
UK	Francis	Frayne	scientist	Institute of Infection and Immunity, School of Medicine, Cardiff University
UK	Jessica	Rees	scientist	Institute of Infection and Immunity, School of Medicine, Cardiff University
UK	Lim	Jones	clinician	Specialist Antimicrobial Chemotherapy Unit, Public Heath Wales, Cardiff, UK
UK	Susanna	Dunachie	scientist	Centre for Tropical Medicine and Global Health, Nuffield Department of Medicine, University of Oxford
UK	Brad	Spiller	scientist	Institute of Infection and Immunity, School of Medicine, Cardiff University
UK	Julian	Parkhill	scientist	Cambridge Infectious Diseases, Department of Veterinary Medicine, Cambridge University
