# Supplementary material for: Characterization of antimicrobial-resistant Gram-negative bacteria that cause neonatal sepsis in seven low- and middle-income countries
Source: Nat Microbiol. 2021 Mar 29;6(4):512–23. doi: 10.1038/s41564-021-00870-7 (PMC8007471; doi:10.1038/s41564-021-00870-7)
Supplement: Supplementary file 2 — Reporting Summary [file 41564_2021_870_MOESM2_ESM.pdf]

nature research

Corresponding author(s): Kirsty M Sands

Last updated by author(s): Dec 6, 2020

Reporting Summary

Nature Research wishes to improve the reproducibility of the work that we publish. This form provides structure for consistency and transparency in reporting. For further information on Nature Research policies, see our Editorial Policies and the Editorial Policy Checklist.

Statistics

For all statistical analyses, confirm that the following items are present in the figure legend, table legend, main text, or Methods section.

n/a	Confirmed	
☐	☒	The exact sample size (n) for each experimental group/condition, given as a discrete number and unit of measurement
☐	☐	A statement on whether measurements were taken from distinct samples or whether the same sample was measured repeatedly
☐	☒	The statistical test(s) used AND whether they are one- or two-sided<br>*Only common tests should be described solely by name; describe more complex techniques in the Methods section.*
☐	☐	A description of all covariates tested
☐	☐	A description of any assumptions or corrections, such as tests of normality and adjustment for multiple comparisons
☐	☒	A full description of the statistical parameters including central tendency (e.g. means) or other basic estimates (e.g. regression coefficient) AND variation (e.g. standard deviation) or associated estimates of uncertainty (e.g. confidence intervals)
☐	☒	For null hypothesis testing, the test statistic (e.g. F, t, r) with confidence intervals, effect sizes, degrees of freedom and P value noted<br>*Give P values as exact values whenever suitable.*
☐	☐	For Bayesian analysis, information on the choice of priors and Markov chain Monte Carlo settings
☐	☐	For hierarchical and complex designs, identification of the appropriate level for tests and full reporting of outcomes
☐	☐	Estimates of effect sizes (e.g. Cohen's d, Pearson's r), indicating how they were calculated

Our web collection on statistics for biologists contains articles on many of the points above.

Software and code

Policy information about availability of computer code

Data collection	No software was used in the data collection. At the low middle income countries, research nurses completed questionnaires with the women approaching labor. These questionnaires were either transcribed onto paper, due to availability of resources/infrastructure, i.e. Internet access, and later uploaded into Bristol Online survey (BOS) or directly entered into BOS using a tablet device provided by the project.
Data analysis	CLIMB (v1.0)<br>Trimgalore (v0.4.3)<br>fastqc (v0.11.2)<br>MultiQC (v1.7)<br>Flash (v1.2.11)<br>SPAdes (v3.9.0)<br>BWA (v.0.7.15)<br>samtools (v1.3.1)<br>Pilon (v1.22)<br>quast (v.2.1)<br>Blast nt (https://blast.ncbi.nlm.nih.gov/Blast.cgi) (v2.2.25)<br>PathogenWatch (v.3.13.10; https://pathogen.watch)<br>srst2 (v0.2.0)<br>ABRicate (v0.9.7)<br>BIGSbd (v1.25.1)<br>Kaptive (v0.7.0)<br>and Kleborate (v0.2.0)<br>SerotypeFinder (v2.0)<br>SeqSero (v1.0)<br>ClermonTyping (v.1.3.0)

Prokka (v1.12)
Roary (v3.12.0)
FastTree (v2.1.11)
iTOL (v5.7)
networkD3 package, Rv3.6.2
Geneious prime (2020.1.2)

For manuscripts utilizing custom algorithms or software that are central to the research but not yet described in published literature, software must be made available to editors and reviewers. We strongly encourage code deposition in a community repository (e.g. GitHub). See the Nature Research guidelines for submitting code & software for further information.

Data

Policy information about availability of data

All manuscripts must include a data availability statement. This statement should provide the following information, where applicable:

- Accession codes, unique identifiers, or web links for publicly available datasets
- A list of figures that have associated raw data
- A description of any restrictions on data availability

Sequences reads were submitted to the European Nucleotide Archive (ENA) and given the project number PRJEB33565. A list of individual accession numbers for 916 Gram-negative bacteria can be found in the source data (to accompany figure 4,5 & 6).

Databases used within this study:
VFDB: http://www.mgc.ac.cn/VFs/download.htm
NCBI: https://github.com/tseemann/abricate/tree/master/db/ncbi
Resfinder: https://github.com/tseemann/abricate/tree/master/db/resfinder
Plasmidfinder: https://bitbucket.org/genomicepidemiology/plasmidfinder/src/master
mlst: https://github.com/tseemann/mlst/tree/master/db/pubmlst
MGE: https://bitbucket.org/mhkj/mge_finder/src/master/me_finder/
Serotype finder: https://bitbucket.org/genomicepidemiology/serotypefinder/src/master
Seqsero: http://www.denglab.info/SeqSero

Previously published datasets downloaded from the ENA repository used for comparative genomics analysis: PRJEB2111, PRJEB2581 and PRJEB20875. Genomes were downloaded from NCBI: PHGE01000000-PHGR01000000, ATNW00000000, ATNV00000000.

Field-specific reporting

Please select the one below that is the best fit for your research. If you are not sure, read the appropriate sections before making your selection.

☒ Life sciences ☐ Behavioural & social sciences ☐ Ecological, evolutionary & environmental sciences

For a reference copy of the document with all sections, see nature.com/documents/nr-reporting-summary-flat.pdf

Life sciences study design

All studies must disclose on these points even when the disclosure is negative.

Sample size	The sampling method was purposive and a formal sample size calculation was not conducted.<br><br>Based on previous studies led by PI Professor Timothy Walsh (unpublished studies/awaiting publication), BARNARDS anticipated the enrollment level between 500-2000 neonates per clinical site for the duration of the study (depending on geographical location i.e. smaller rural site would have a smaller catchment area). With the anticipated sepsis rate (based on limited public accessible data), this would capture between 1,000-2,500 bacterial (both Gram-negative and Gram-positive) blood culture positive sepsis cases across seasonal variation with an estimated 1,000 Gram-negative sepsis cases.
Data exclusions	The following exclusion criteria was pre-defined: the sepsis case infant/mother sampling pair was excluded in the case of a still born. Following this, data was retrospectively excluded based on the following criteria:<br>- Incomplete questionnaire; missing multiple data points in the epidemiological dataset<br>- Mother asked for infant withdrawal<br>- Error/substantial inconsistencies in the questionnaire - laboratory sampling match up.
Replication	N/A - Findings were not replicated as this manuscript is a full genomics characterisation of sepsis causing bacteria from low middle income countries (LMICs). All viable isolates eligible for sequencing were included into the analysis.
Randomization	Randomization was not relevant to the study. All women approaching labor were (following consenst) enrolled onto the study. If the neonate/infant presented with sepsis, the infant was also enrolled to allow a blood culture to be taken.
Blinding	Blinding was not necessary for this study as we were characterizing all blood culture isolates along with the corresponding infant's clinical data.

Reporting for specific materials, systems and methods

We require information from authors about some types of materials, experimental systems and methods used in many studies. Here, indicate whether each material, system or method listed is relevant to your study. If you are not sure if a list item applies to your research, read the appropriate section before selecting a response.

Materials & experimental systems

n/a	Involved in the study
☒	☐ Antibodies
☒	☐ Eukaryotic cell lines
☒	☐ Palaeontology and archaeology
☒	☐ Animals and other organisms
☐	☒ Human research participants
☒	☐ Clinical data
☒	☐ Dual use research of concern

Methods

n/a	Involved in the study
☒	☐ ChIP-seq
☒	☐ Flow cytometry
☒	☐ MRI-based neuroimaging

Human research participants

Policy information about studies involving human research participants

Population characteristics	BARNARDS was a multi-site international prospective observational study incorporating two recruitment pathways:<br>i.) Birth-Cohort: All mothers in labour admitted to clinical-sites were recruited prospectively and their infant(s) followed up until 60-days old or death.<br>ii.) Infant Admissions (IA): Infant(s) admitted to clinical-sites showing signs of suspected sepsis in the first 60-days of life until 60- days old or death.<br>2<br>nature research | reporting summary October 2018<br><br>For this study, isolates recovered from blood cultures were included into the characterisation of infant sepsis irrespective of cohort pathway.<br>General population characteristics of the mothers' (oustide of the scope of this manuscript): <10% previously had stillbirth, approx. 25% were first time mothers', 75% were aged between 21-35 years old. Infants' presenting with sepsis were followed up for 60 days of life. Onset of sepsis was recorded, early onset (EOS) <72h, and late onset (LOS) >72h.
Recruitment	BARNARDS recruited from 12 clinical sites from Rwanda, Bangladesh, Ethiopia, Nigeria, Pakistan, India and South Africa. Where possible, large public hospitals were chosen.<br><br>Following the presentation of information regarding the study, mothers in labour were enrolled into the study. Consent was collected by trained research staff and using local languages. Corresponding neonates presenting with clinical signs of sepsis were then enrolled into the study.<br>Additionally, neonates not born within the clinical sites that were admitted with clinical signs of sepsis were also enrolled into the study following consent from the mother. The corresponding mothers were also enrolled into the study for the collection of demographic data.<br>Neonatal follow-up was carried out at day 3, 7, 14, 28, and 60 by research nurses either face-to-face or by telephone. Neonates remained in the study until 60 days old, withdrawal, or death.<br>This study incorporated two recruitment pathways to include both neonates born within the clinical sites, and also neonates in the larger catchment areas presenting to the hospital with signs of sepsis.
Ethics oversight	Site committees Named PI Reference(s) Approval date(s)<br>BC - Ethical Review Committee, Bangladesh Institute of Child Health Samir Kumar Saha BICH-ERC-4/3/2015 15/09/2015<br>BK - Ethical Review Committee, Bangladesh Institute of Child Health Samir Kumar Saha BICH-ERC-4/3/2015 15/09/2015<br>ES - Boston Children's Hospital Grace Chan IRB-P00023058 11/08/2016<br>IN - Institutional Ethics Committee, National Institute of Cholera and Enteric Diseases and Institue of Post Graduate Medical Education and Research, IPGME&R Research Oversight Committee Sulagna Basu A-I/2016-IEC and Inst/IEC/2016/508 17/11/2016 and 04/11/2016<br>NK - Kano State Hospitals Management Board Kenneth Iregbu 8/10/1437AH 13/07/2016<br>NN - Health Research Ethics Committee (HREC), National Hospital, Abuja Kenneth Iregbu NHA/EC/017/2015 27/04/2015<br>NW - Health Research Ethics Committee (HREC), National Hospital, Abuja Kenneth Iregbu NHA/EC/017/2015 27/04/2015<br>PC - Shaheed Zulfiqar Ali Bhutto Medical University, Pakistan Institute of Medical Sciences (PIMS) Islamabad Rabaab Zahra NA, signed letter from Prof. Tabish Hazir 27/05/2015<br>PP - Shaheed Zulfiqar Ali Bhutto Medical University, Pakistan Institute of Medical Sciences (PIMS) Islamabad Rabaab Zahra NA, signed letter from Prof. Tabish Hazir 27/05/2015<br>RK - Republic of Rwanda, National Ethics Committee Jean-Baptiste Mazarati No342/RNEC/2015 10/11/2015<br>RU - Republic of Rwanda, National Ethics Committee Jean-Baptiste Mazarati No342/RNEC/2015 10/11/2015<br>ZAT - Stellenbosch University and Tygerberg Hospital, Research projects, Western Cape Government Shaheen Mehtar N15/07/063 04/12/2015 and 02/02/2016

Note that full information on the approval of the study protocol must also be provided in the manuscript.
